# Supplementary figures and images for: Shortcut citations in the methods section: Frequency, problems, and strategies for responsible reuse
Source: PLoS Biol. 2024 Apr 2;22(4):e3002562. doi: 10.1371/journal.pbio.3002562 (PMC10986953; doi:10.1371/journal.pbio.3002562)

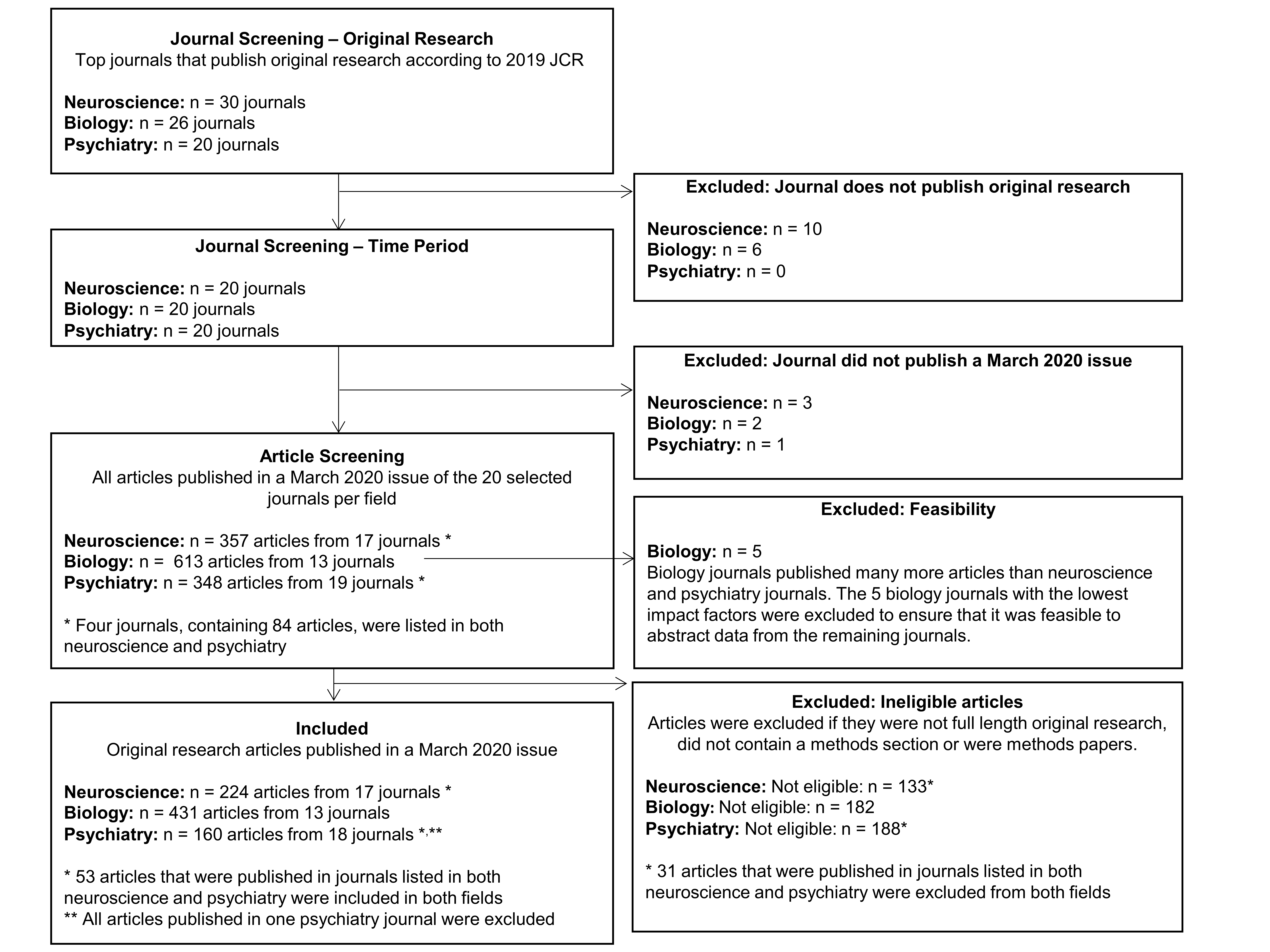

Supplement: S1 Fig — This flow chart illustrates the journal and article screening process and shows the number of observations excluded and reasons for exclusion at each phase of screening. Data are available at https://osf.io/d2sa3/, in the methodological citations study folder [12]. (TIF) [file pbio.3002562.s001.tif]

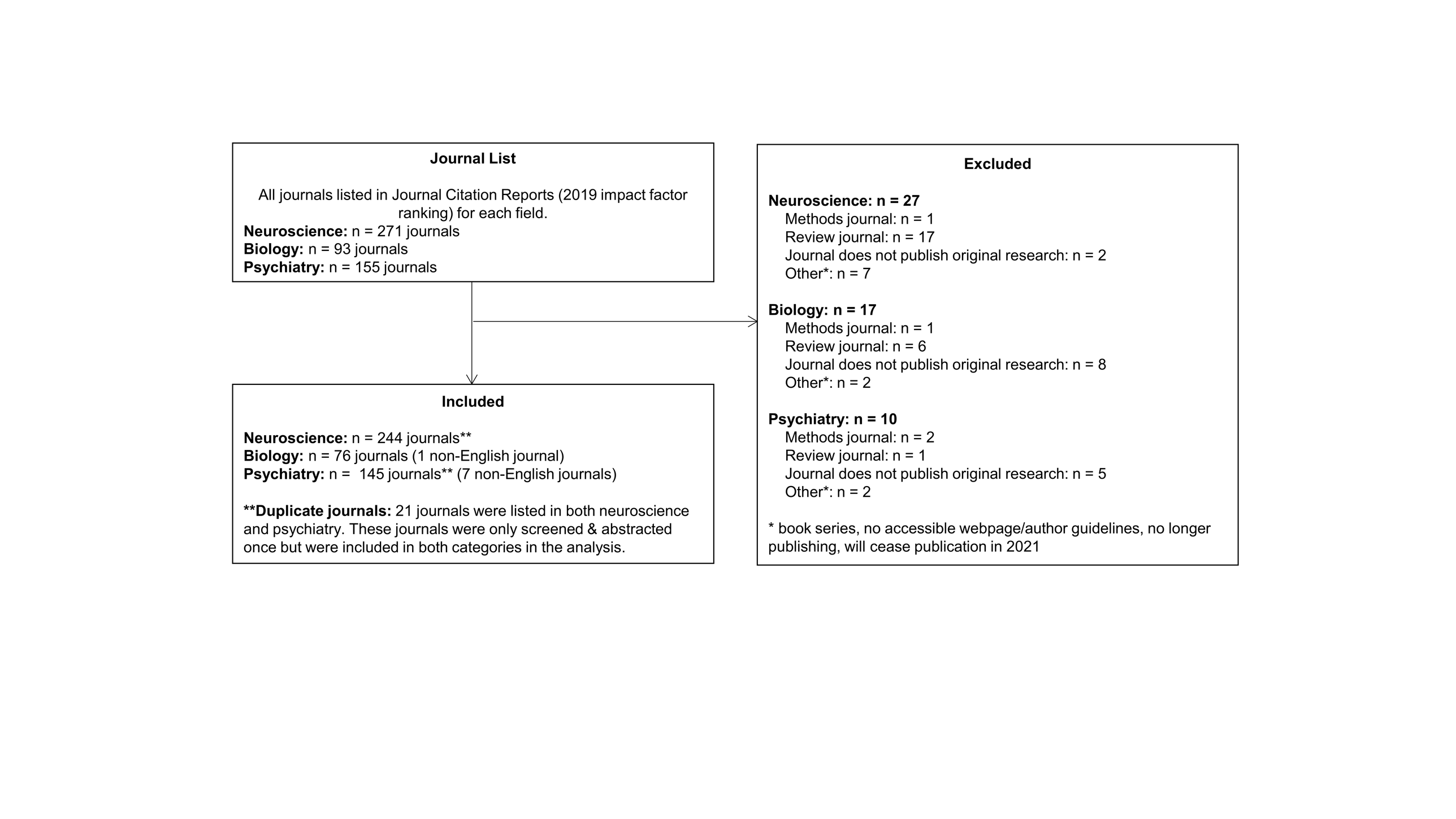

Supplement: S4 Fig — This flow chart illustrates the journal screening process and shows the number of observations excluded and reasons for exclusion at each phase of screening. Data are available at https://osf.io/d2sa3/, in the journal policy study folder [12]. (TIF) [file pbio.3002562.s004.tif]
