# Supplementary material for: Shortcut citations in the methods section: Frequency, problems, and strategies for responsible reuse
Source: PLoS Biol. 2024 Apr 2;22(4):e3002562. doi: 10.1371/journal.pbio.3002562 (PMC10986953; doi:10.1371/journal.pbio.3002562)

Neuroscience 1st quintile

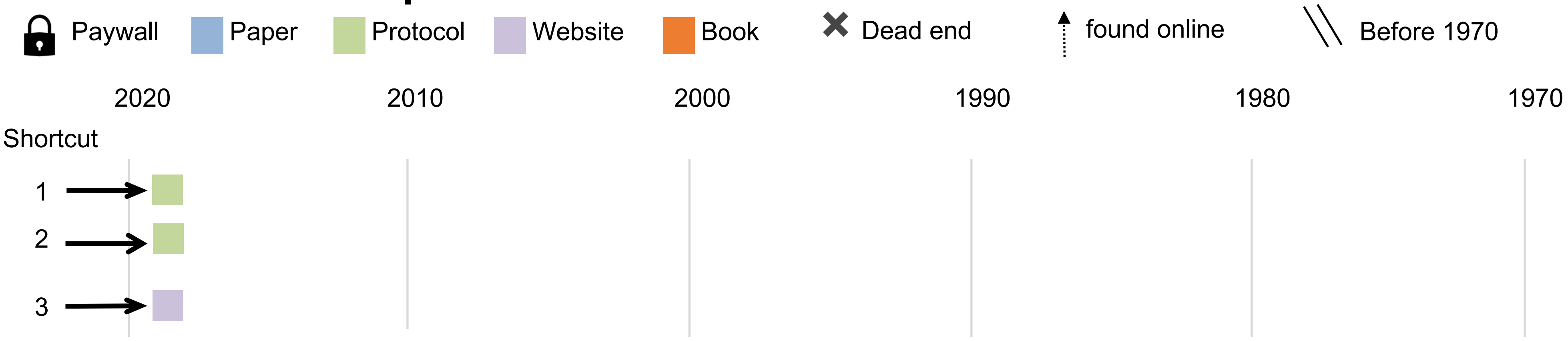

Neuroscience 2nd quintile

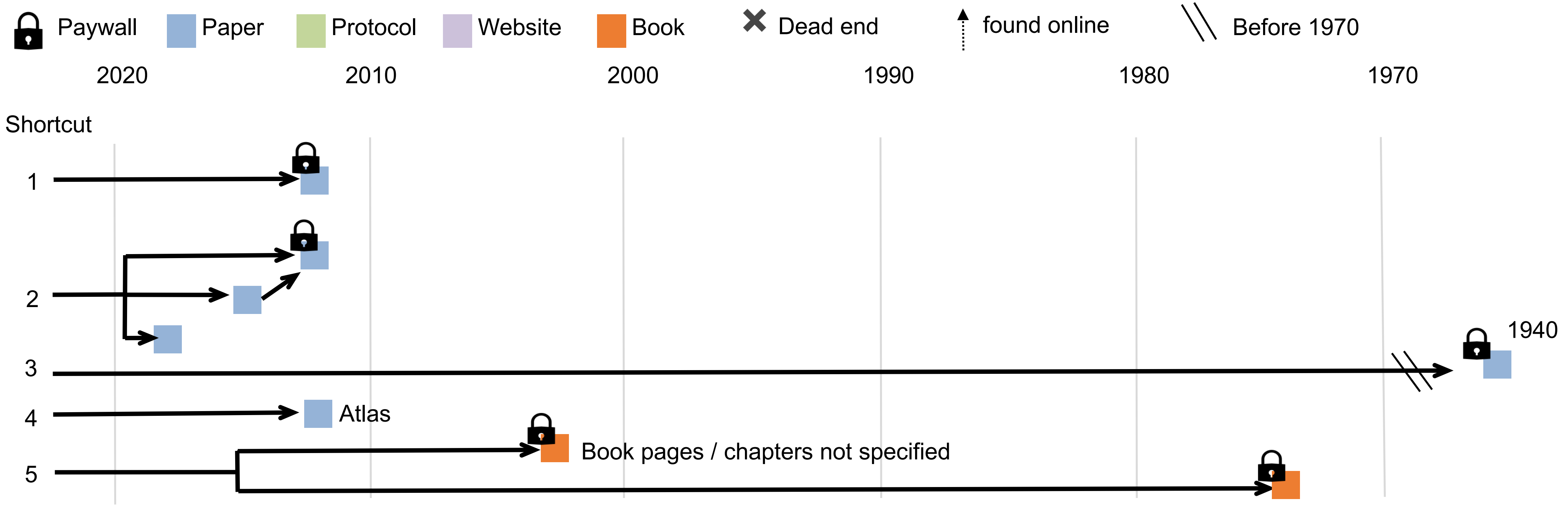

Neuroscience 3rd quintile

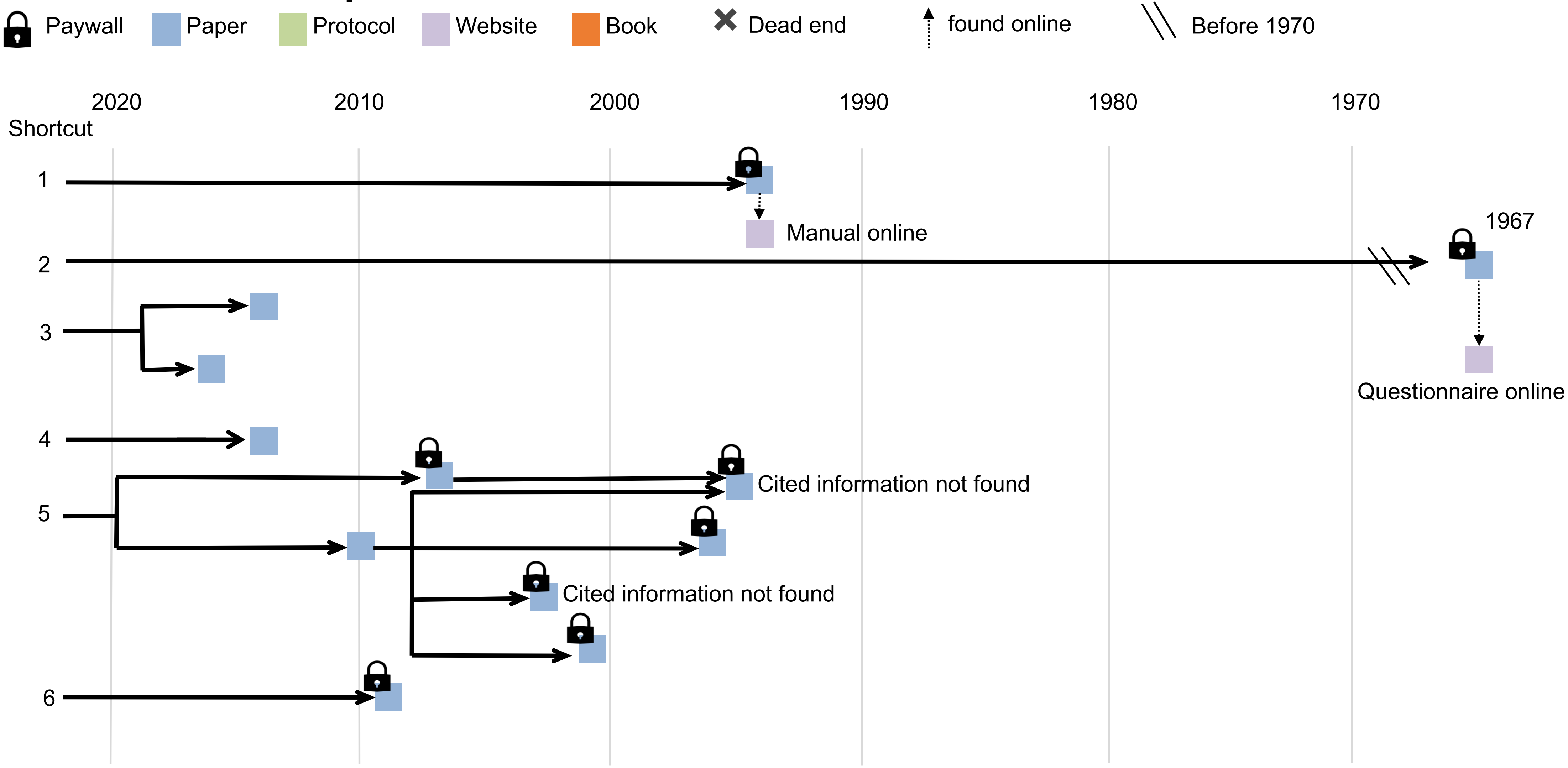

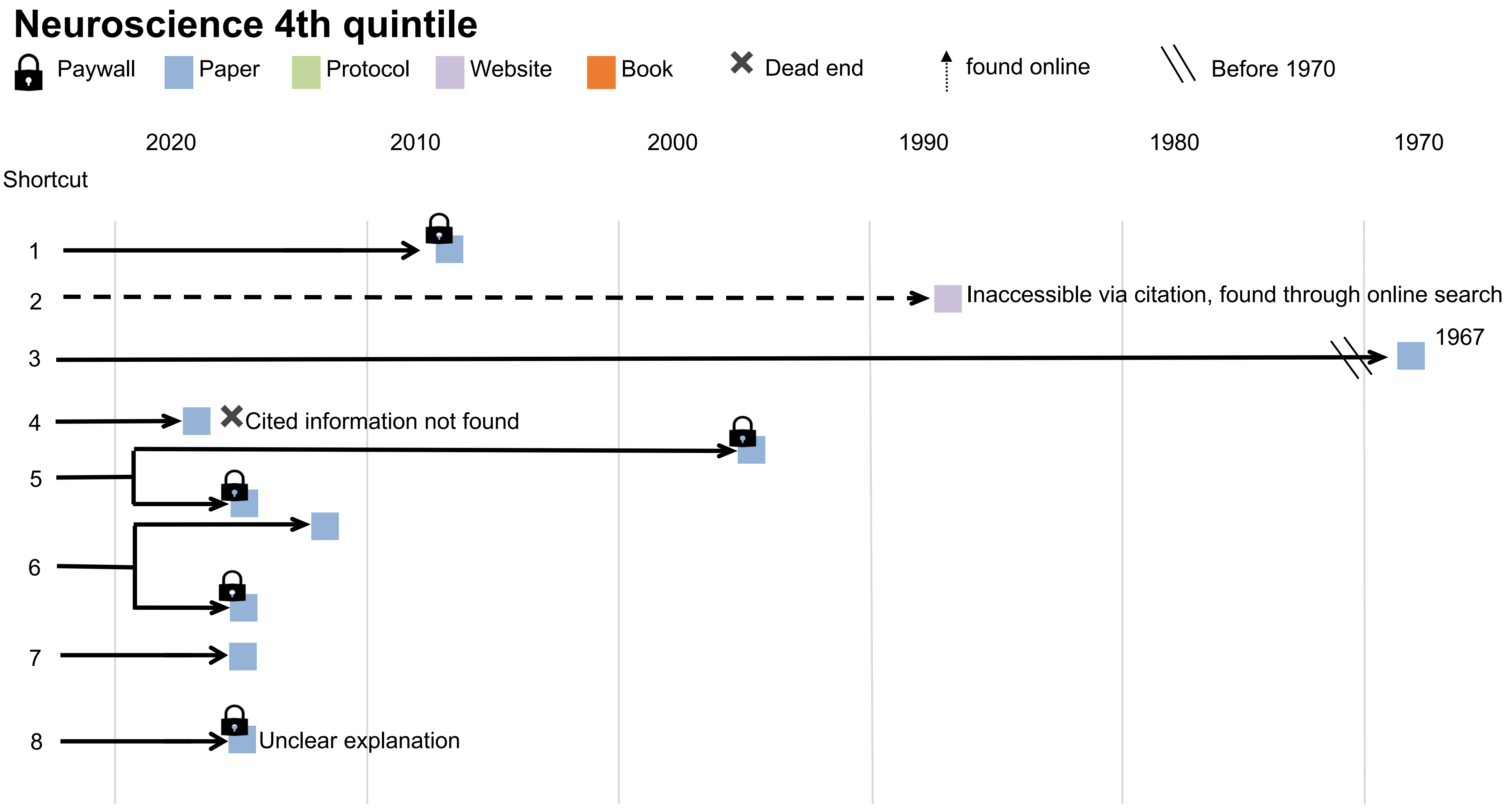
Neuroscience 4th quintile
Paywall
Paper
Protocol
Website
Book
Dead end
found online
Before 1970
2020
2010
2000
1990
1980
1970
Shortcut
1
2
Inaccessible via citation, found through online search
3
1967
4
Cited information not found
5
6
7
8
Unclear explanation

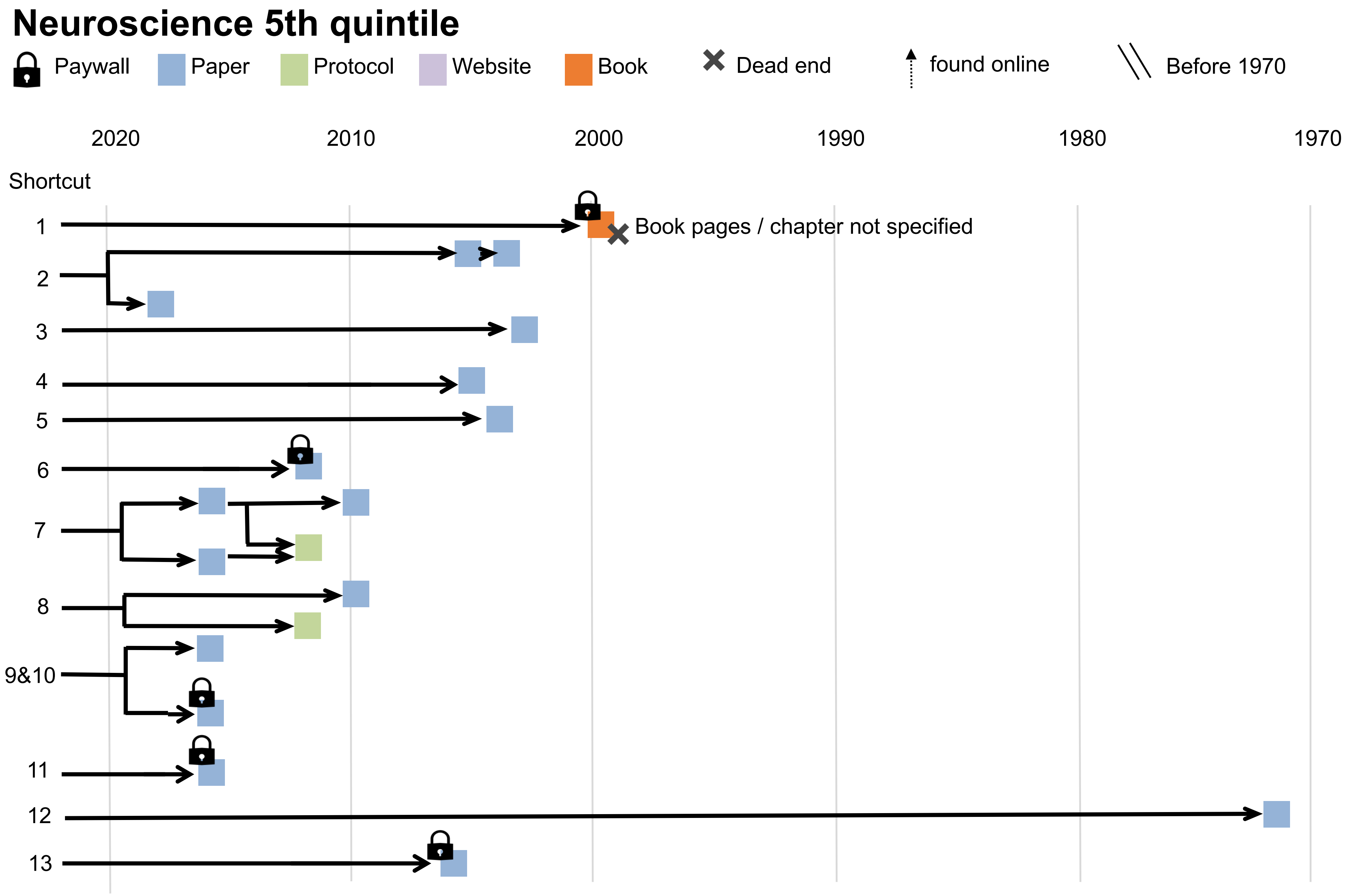
Neuroscience 5th quintile
Paywall
Paper
Protocol
Website
Book
Dead end
found online
Before 1970
2020
2010
2000
1990
1980
1970
Shortcut
1
2
3
4
5
6
7
8
9&10
11
12
13
Book pages / chapter not specified

Biology 1st quintile

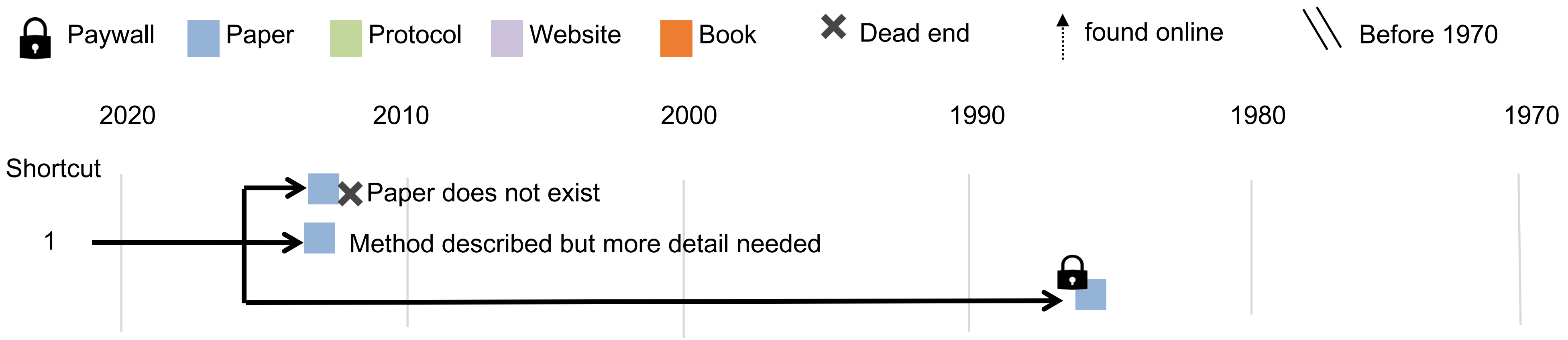

Biology 2nd quintile

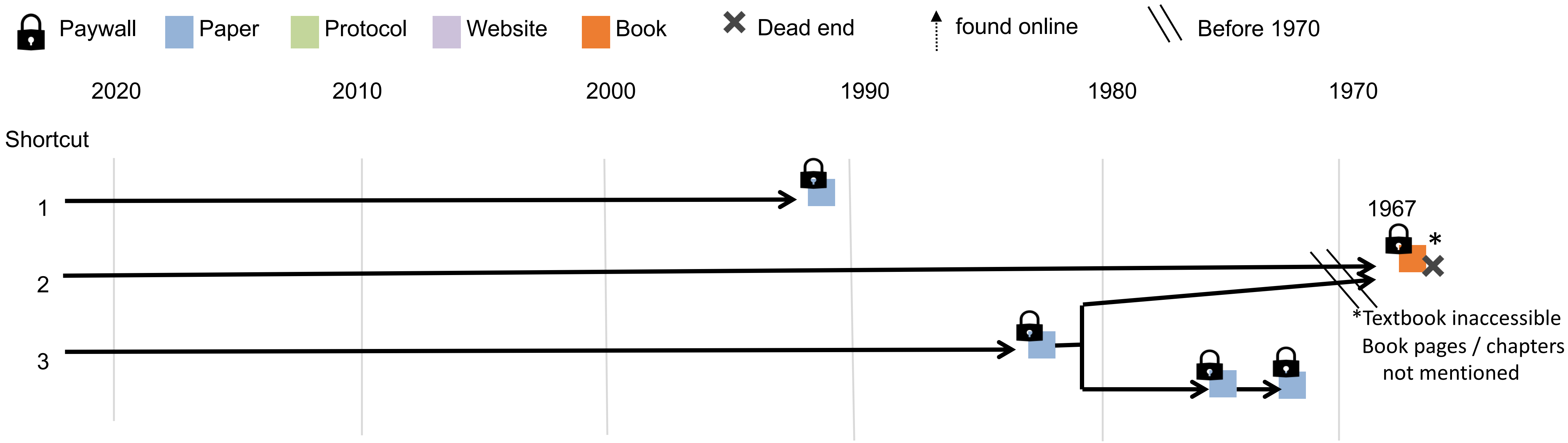

Biology 3rd quintile

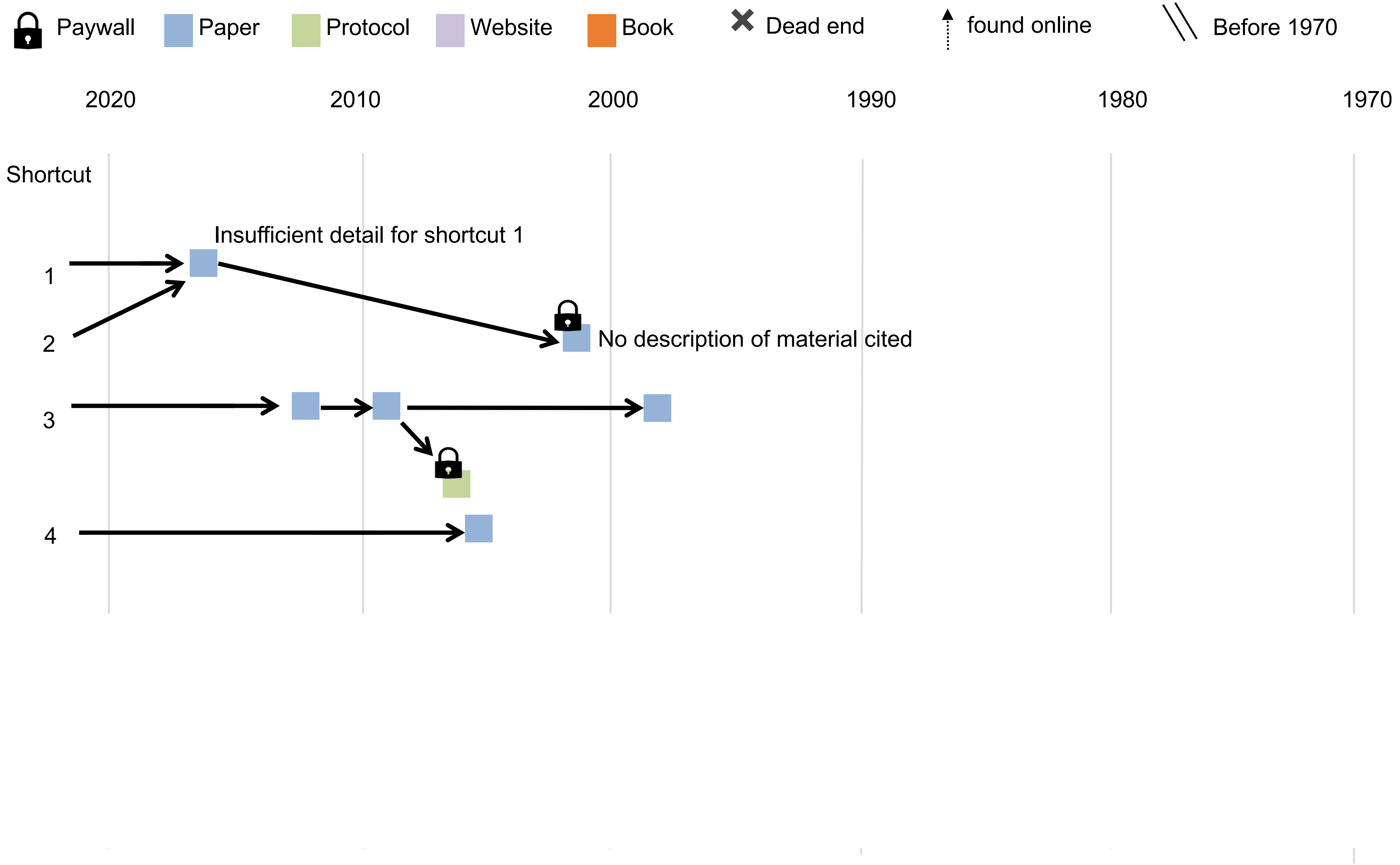

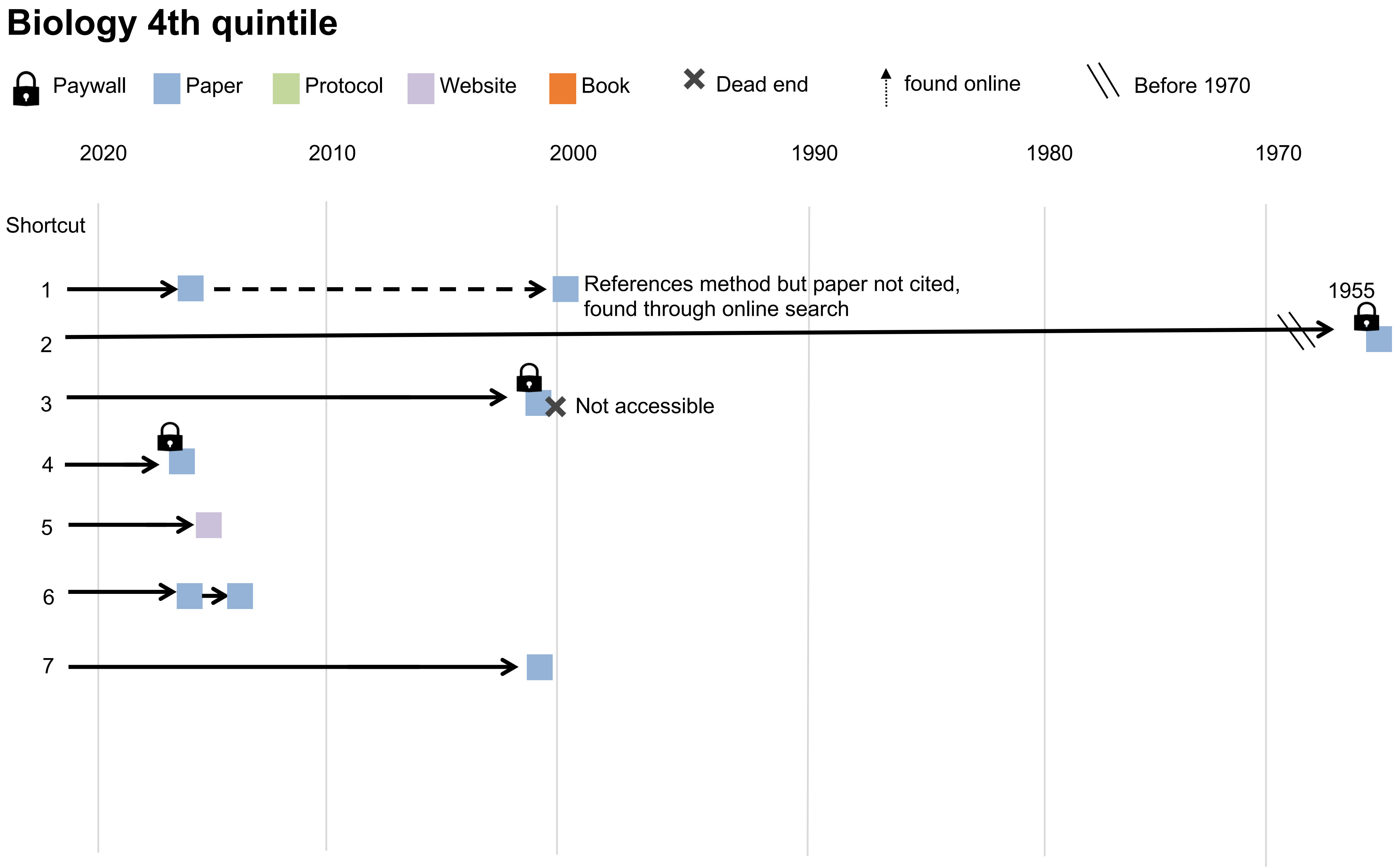

Biology 4th quintile
Paywall
Paper
Protocol
Website
Book
Dead end
found online
Before 1970
2020
2010
2000
1990
1980
1970
Shortcut
1
2
3
4
5
6
7
References method but paper not cited,
found through online search
1955
Not accessible

Biology 5th quintile

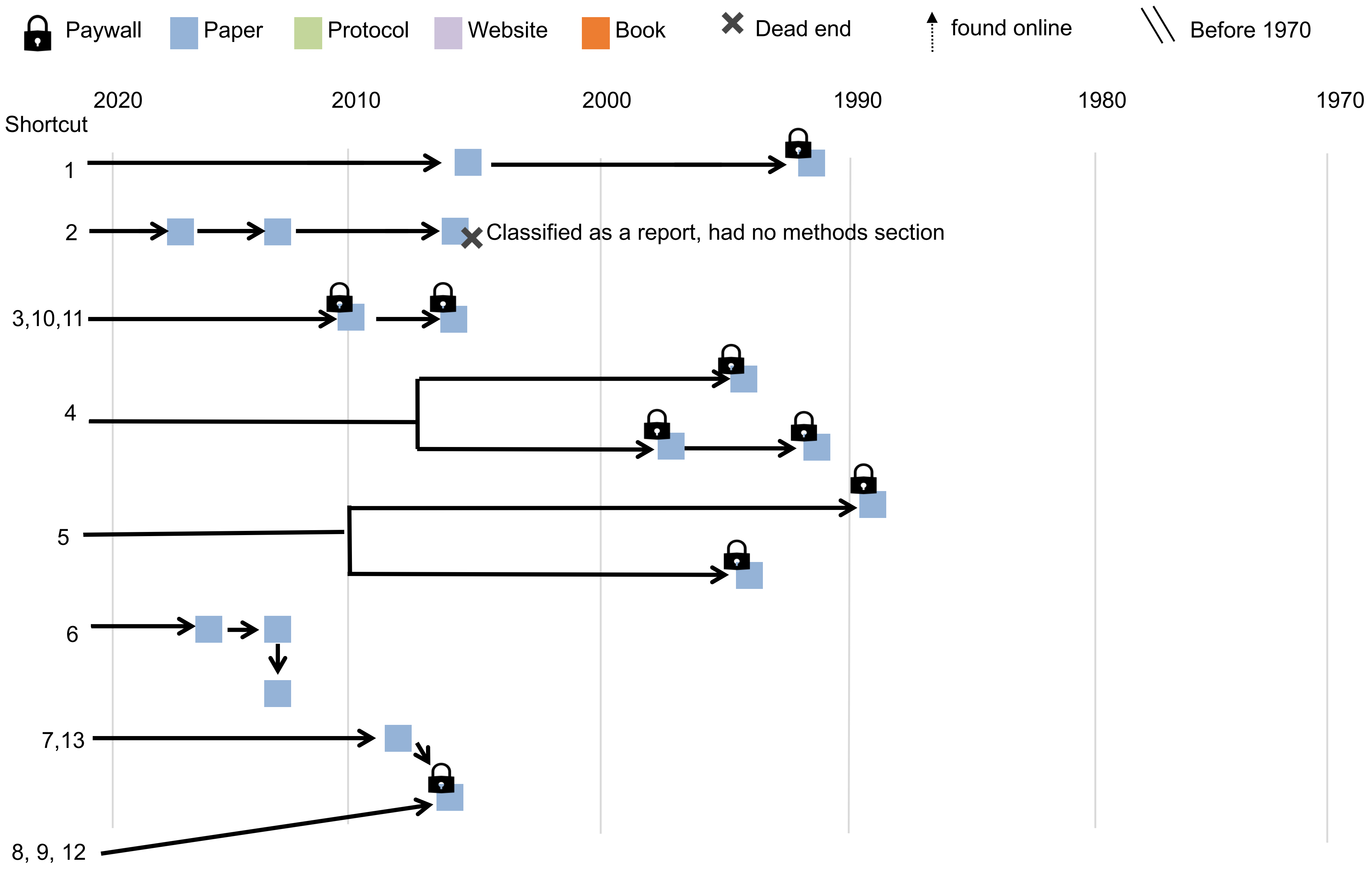

Psychiatry 1st quintile

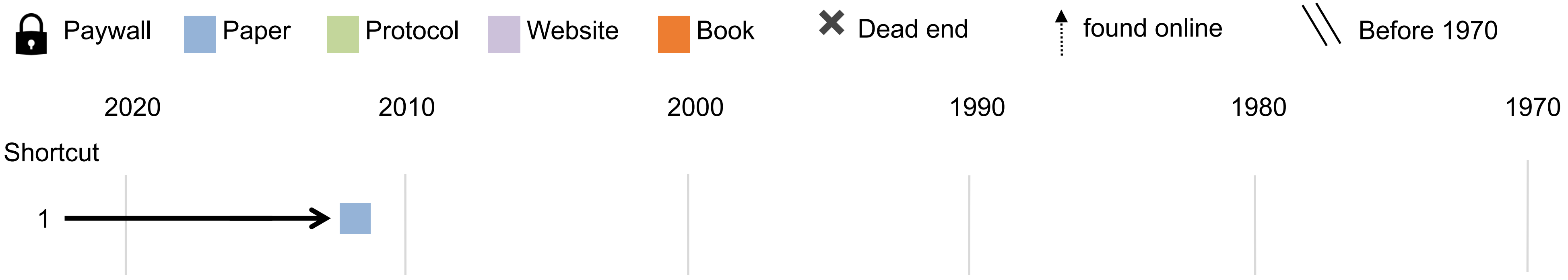

Psychiatry 2nd quintile

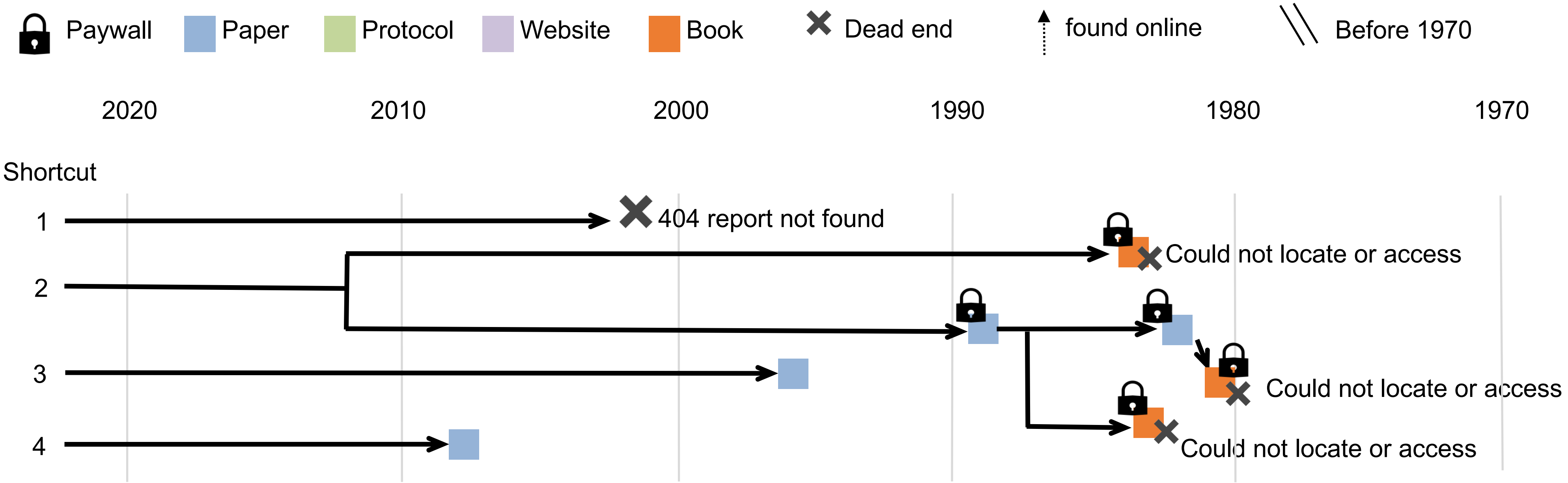

Psychiatry 3rd quintile

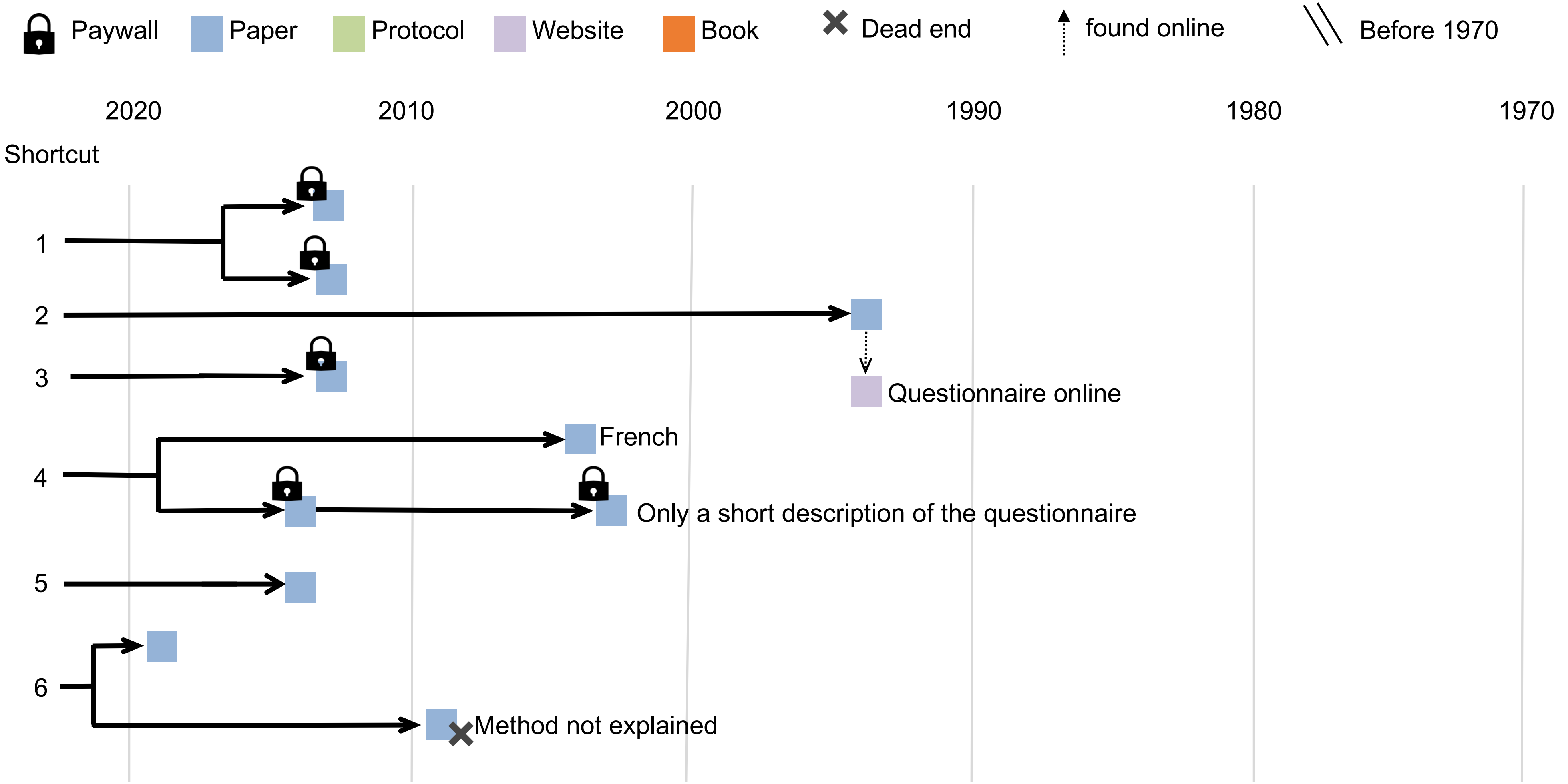

Psychiatry 4th quintile

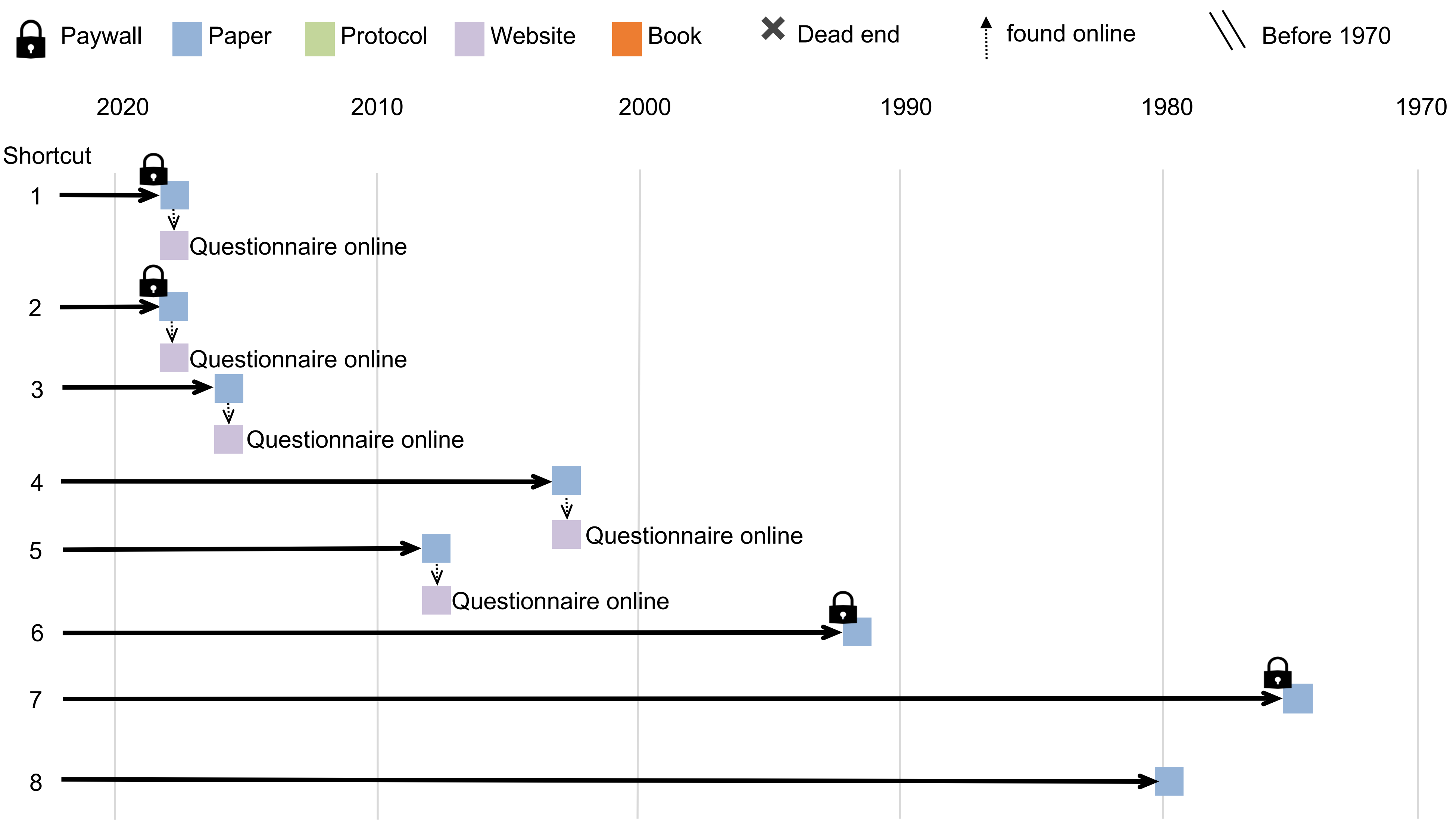

Psychiatry 5th quintile

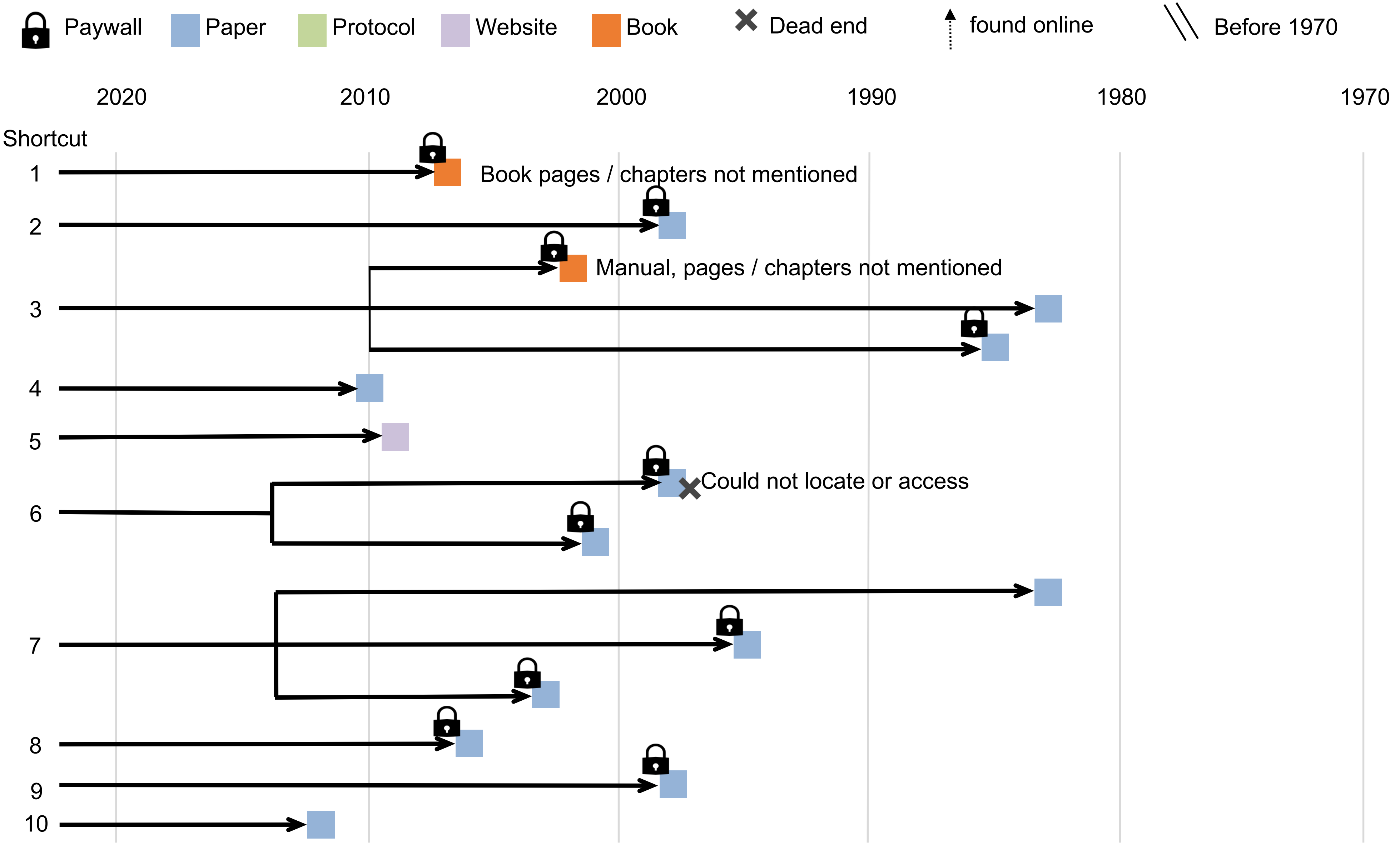

Supplement: S3 Fig — These diagrams map the process of finding methodological details for each of the 15 papers in the shortcut citation chains study. Reviewers consulted resources cited in shortcut citations to find methodological details. The diagrams show the publication year and type of each cited resource and whether the resource was behind a paywall. Chains of shortcut citations occur when the cited source also uses a methodological shortcut citation to describe the method. Text on the diagram provides information describes problems encountered when searching for details about the cited method. (PDF) [file pbio.3002562.s003.pdf]
