# Supplementary material for: Shortcut citations in the methods section: Frequency, problems, and strategies for responsible reuse
Source: PLoS Biol. 2024 Apr 2;22(4):e3002562. doi: 10.1371/journal.pbio.3002562 (PMC10986953; doi:10.1371/journal.pbio.3002562)
Supplement: S1 Table — Values are n, or n (% of all articles). Screening was performed to exclude articles that were not full-length original research articles (e.g., reviews, editorials, perspectives, commentaries, letters to the editor, short communications), were not published in March 2020, or did not have a methods section. No issue indicates that the journal did not publish an issue or any articles in March 2020. Data are available at https://osf.io/d2sa3/, in the methodological citations study folder [12]. * Journals were included on both the neuroscience and psychiatry (S3 Table) lists. (DOCX) [file pbio.3002562.s005.docx]

| **S1 Table:** Number of articles examined for each neuroscience journal | | |
| --- | --- | --- |
| **Journal** | **Articles Screened**  (n = 357) | **Articles Included**  (n = 224, 63%) |
| Nature Neuroscience | 18 | 13 (72%) |
| Journal of Pineal Research | 8 | 8 (100%) |
| Neuron | 34 | 20 (59%) |
| Acta Neuropathologica | 12 | 9 (75%) |
| Molecular Psychiatry* | 18 | 10 (56%) |
| Nature Human Behavior | 19 | 8 (42%) |
| Biological Psychiatry* | 27 | 16 (59%) |
| Brain | 34 | 18 (53%) |
| Molecular Neurodegeneration | 8 | 5 (63%) |
| Progress in Neurobiology | 4 | 3 (75%) |
| Annals of Neurology | 16 | 13 (81%) |
| Neurology – Neuroimmunology & Neuroinflammation | 25 | 17 (68%) |
| Neuropathology and Applied Neurobiology | no issue |  |
| Neurobiology of Stress | no issue |  |
| Neuropsychopharmacology* | 18 | 13 (82%) |
| NPJ Parkinson’s Disease | 0 | 0 (0%) |
| Brain, Behavior and Immunity* | 21 | 14 (67%) |
| Brain Stimulation | 62 | 32 (52%) |
| Acta Neuropathologica Communications | 16 | 11 (69%) |
| Alzheimers Research & Therapy | 17 | 14 (82%) |
| Values are n, or n (% of all articles). Screening was performed to exclude articles that were not full-length original research articles (e.g. reviews, editorials, perspectives, commentaries, letters to the editor, short communications, etc.), were not published in March 2020, or did not have a methods section. No issue indicates that the journal did not publish an issue or any articles in March 2020.  * Journals were included on both the neuroscience and psychiatry (Table S3) lists. | | |
