# Supplementary material for: Shortcut citations in the methods section: Frequency, problems, and strategies for responsible reuse
Source: PLoS Biol. 2024 Apr 2;22(4):e3002562. doi: 10.1371/journal.pbio.3002562 (PMC10986953; doi:10.1371/journal.pbio.3002562)
Supplement: S2 Table — Values are n, or n (% of all articles). Screening was performed to exclude articles that were not full-length original research articles (e.g., reviews, editorials, perspectives, commentaries, letters to the editor, short communications), were not published in March 2020, or did not have a methods section. No issue indicates that the journal did not publish an issue or any articles in March 2020. Data are available at https://osf.io/d2sa3/, in the methodological citations study folder [12]. (DOCX) [file pbio.3002562.s006.docx]

| **S2 Table:** Number of articles examined for each biology journal | | |
| --- | --- | --- |
| **Journal** | **Articles Screened**  (n = 613) | **Articles Included**  (n = 431, 70%) |
| Current Biology | 70 | 23 (33%) |
| eLife | 165 | 129 (78%) |
| PLoS Biology | 44 | 28 (64%) |
| BMC Biology | 16 | 13 (81%) |
| Philosophical Transactions of the Royal Society B - Biological Sciences | 52 | 24 (46%) |
| FASEB Journal | 89 | 82 (92%) |
| Bioelectrochemistry | no issue |  |
| Proceedings of the Royal Society B-Biological Sciences | 45 | 40 (89%) |
| Science China – Life Sciences | 17 | 8 (47%) |
| Geobiology | 7 | 7 (100%) |
| Communications Biology | 56 | 51 (91%) |
| Astrobiology | 8 | 8 (100%) |
| Biology | 22 | 15 (68%) |
| Yale Journal of Biology and Medicine | 22 | 3 (14%) |
| Interface Focus | no issue |  |
| Values are n, or n (% of all articles). Screening was performed to exclude articles that were not full-length original research articles (e.g. reviews, editorials, perspectives, commentaries, letters to the editor, short communications, etc.), were not published in March 2020, or did not have a methods section. No issue indicates that the journal did not publish an issue or any articles in March 2020. | | |
