# Supplementary material for: Shortcut citations in the methods section: Frequency, problems, and strategies for responsible reuse
Source: PLoS Biol. 2024 Apr 2;22(4):e3002562. doi: 10.1371/journal.pbio.3002562 (PMC10986953; doi:10.1371/journal.pbio.3002562)
Supplement: S3 Table — Values are n, or n (% of all articles). Screening was performed to exclude articles that were not full-length original research articles (e.g., reviews, editorials, perspectives, commentaries, letters to the editor, short communications), were not published in March 2020, or did not have a methods section. No issue indicates that the journal did not publish an issue or any articles in March 2020. Data are available at https://osf.io/d2sa3/, in the methodological citations study folder [12]. * Journals were included on both the neuroscience and psychiatry (S1 Table) lists. (DOCX) [file pbio.3002562.s007.docx]

| **S3 Table:** Number of articles examined for each psychiatry journal | | |
| --- | --- | --- |
| **Journal** | **Articles Screened**  (n = 348) | **Articles Included**  (n = 160, 46%) |
| World Psychiatry | no issue |  |
| JAMA Psychiatry | 10 | 7 (70%) |
| Lancet Psychiatry | 31 | 3 (10%) |
| Psychotherapy and Psychosomatics | 13 | 1 (8%) |
| American Journal of Psychiatry | 17 | 5 (29%) |
| Molecular Psychiatry* | 18 | 10 (56%) |
| Biological Psychiatry* | 27 | 16 (59%) |
| Journal of Neurology, Neurosurgery and Psychiatry | 21 | 9 (43%) |
| Schizophrenia Bulletin | 28 | 17 (61%) |
| British Journal of Psychiatry | 16 | 5 (31%) |
| Journal of Child Psychology and Psychiatry | 19 | 0 (0%) |
| Journal of the American Academy of Child and Adolescent Psychiatry | 18 | 9 (50%) |
| Neuropsychopharmacology* | 18 | 13 (72%) |
| Brain, Behavior and Immunity* | 21 | 14 (67%) |
| Addiction | 29 | 14 (48%) |
| Epidemiology and Psychiatric Sciences | 4 | 2 (50%) |
| Psychological Medicine | 17 | 13 (76%) |
| Clinical Psychological Science | 15 | 9 (60%) |
| Bipolar Disorders | 15 | 6 (40%) |
| Acta Psychiatrica Scandinavica | 11 | 7 (64%) |
| Values are n, or n (% of all articles). Screening was performed to exclude articles that were not full-length original research articles (e.g. reviews, editorials, perspectives, commentaries, letters to the editor, short communications, etc.), were not published in March 2020, or did not have a methods section. No issue indicates that the journal did not publish an issue or any articles in March 2020.  * Journals were included on both the neuroscience (Table S1) and psychiatry lists. | | |
