# Supplementary material for: Shortcut citations in the methods section: Frequency, problems, and strategies for responsible reuse
Source: PLoS Biol. 2024 Apr 2;22(4):e3002562. doi: 10.1371/journal.pbio.3002562 (PMC10986953; doi:10.1371/journal.pbio.3002562)
Supplement: S4 Table — Values are n (% of articles). Data are available at https://osf.io/d2sa3/, in the methodological citations study folder [12]. (DOCX) [file pbio.3002562.s008.docx]

| **S4 Table:** Methods repositories used in neuroscience, biology and psychiatry | | | |
| --- | --- | --- | --- |
| **Repository** | **Neuroscience**  (n = 224 articles) | **Biology**  (n = 431 articles) | **Psychiatry**  (n = 160 articles) |
| GitHub | 18 (8%) | 33 (8%) | 2 (1%) |
| ClinicalTrials.gov | 11 (5%) | 1 (0.2%) | 7 (4%) |
| Open Science Framework | 2 (1%) | 4 (1%) | 2 (1%) |
| Mendeley | 2 (1%) | 2 (0.5%) | 1 (0.5%) |
| FigShare | 0 (0%) | 7 (2%) | 0 (0%) |
| Dryad | 0 (0%) | 5 (1%) | 0 (0%) |
| Gene Expression Omnibus | 2 (1%) | 1 (0.2%) | 0 (0%) |
| UMIN-CTR | 1 (0.5%) | 0 (0%) | 1 (0.5%) |
| Clinicaltrialsregister.eu | 1 (0.5%) | 0 (0%) | 1 (0.5%) |
| Values are n (% of articles). | | | |
