# Supplementary material for: Shortcut citations in the methods section: Frequency, problems, and strategies for responsible reuse
Source: PLoS Biol. 2024 Apr 2;22(4):e3002562. doi: 10.1371/journal.pbio.3002562 (PMC10986953; doi:10.1371/journal.pbio.3002562)
Supplement: S5 Table — This table shows summary statistics for Fig 3A. IQR, interquartile range. Data are available at https://osf.io/d2sa3/, in the methodological citations study folder [12]. (DOCX) [file pbio.3002562.s009.docx]

**Table S5:** Age of youngest, median and oldest shortcut citations within a paper for each field

| **Citation age** | **Field** | **Probable shortcut citations**  Median (IQR) in years | **Possible shortcut citations**  Median (IQR) in years |
| --- | --- | --- | --- |
| Youngest | Neuroscience | 4 (2, 7) | 3 (2, 6) |
|  | Biology | 3 (2, 6) | 4 (2, 7) |
|  | Psychiatry | 4 (3, 8) | 5 (3, 8) |
| Median | Neuroscience | 9 (5.5, 15) | 7 (4, 10.5) |
|  | Biology | 7 (4, 11) | 6 (4, 10) |
|  | Psychiatry | 11.75 (6.5, 13.375) | 9 (5.5, 15) |
| Oldest | Neuroscience | 20 (11, 35) | 14 (7, 22) |
|  | Biology | 13 (7, 22) | 9 (5, 16) |
|  | Psychiatry | 24 (12, 34) | 19 (11, 30) |

This table shows summary statistics for Figure 3a.

Abbreviations: IQR, interquartile range.
