# Supplementary material for: Shortcut citations in the methods section: Frequency, problems, and strategies for responsible reuse
Source: PLoS Biol. 2024 Apr 2;22(4):e3002562. doi: 10.1371/journal.pbio.3002562 (PMC10986953; doi:10.1371/journal.pbio.3002562)
Supplement: S6 Table — JOVE, Journal of Visualized Experiments; N/A, not applicable; OA, open access. * protocols.io offers a partnership with PLOS ONE where authors can publish a methods article linked to their protocol. (DOCX) [file pbio.3002562.s010.docx]

**Table S6:** Comparing Methods of sharing detailed protocols

|  | **Shortcut citation** | **Supplemental files of paper** | **Protocol repository** | **Protocol journal** |
| --- | --- | --- | --- | --- |
| Examples | N/A | N/A | protocols.io  Protocol Exchange  ClinicalTrials.gov (clinical trials)  PROSPERO (systematic  review pre-registration) | **Online:** Bio-protocol.org  **Print:** Nature Protocols, “Current Protocols in …”  series  **Video:** JOVE |
| Structured format | N/A | No  No consistency in what is reported; typically follows the overview format of methods sections; step-by-step protocols are rare | Yes  Platforms differ in the level of detail required (essential information vs. comprehensive, step-by-step protocols) | Yes  Step-by-step protocols  Video protocols |
| Tracks protocol evolution (Are updates possible?) | Citing author must specify modifications to cited methods | **Static:** Can’t be updated after publication | **Living protocol:** Authors can quickly share updated versions. Repositories such as protocols.io allow authors to share forked versions specifying their modifications of someone else’s protocol. Versioning and forking allow scientists to track changes over time, both within and across labs. | **Static:** Reflects what one lab is doing at a single point in time |
| Peer reviewed | Cited resource may or may not be peer reviewed | The quality of peer-review for supplements may be variable | No, but versions or forks indicate re-use  * | Yes |
| Effort required | Low | Low to medium | Medium | High |
| DOI citable | N/A | Yes (DOI for paper) | Yes | Yes |
| Findable | N/A | No  Search engines can only find the paper, which may or may not include supplements | Yes  Scientists can search protocol repositories | Yes  Indexed in major publication search engines |
| Open access (OA) | Cited resource may or may not be OA | Depends on the journal / publisher. Some paywalled publishers offer free access to supplements; others don’t. Supplemental files aren’t consistently shared on publication repositories or through interlibrary loan programs. | OA | Depends on the journal and article |
| Other considerations | Publications with detailed methods descriptions that are similar to the authors’ protocols may not be available |  | Some repositories can be used to share protocols at any time, whereas others are designed to pre-register protocols before the study begins. | Often used to share and obtain credit for new, innovative methods  Some journals publish study protocols for clinical studies, systematic reviews, etc. |
| Abbreviations: JOVE, Journal of Visualized Experiments; N/A, not applicable; OA, open access  *protocols.io offers a partnership with PLOS One where authors can publish a methods article linked to their protocol | | | | |
