# Supplementary material for: Shortcut citations in the methods section: Frequency, problems, and strategies for responsible reuse
Source: PLoS Biol. 2024 Apr 2;22(4):e3002562. doi: 10.1371/journal.pbio.3002562 (PMC10986953; doi:10.1371/journal.pbio.3002562)
Supplement: Attachment — Submitted filename: Shortcut_citations_R2R_20231014.docx [file pbio.3002562.s011.docx]

***Reviewer #1 Comment:*** *Summary:

The manuscript addresses the question of "shortcut citations" in methods description. Although this problem is frequently mentioned in debates about methodological reproducibility, it is understudied and it's nice to see actual research about it.
The results contain three main sections, which study (a) the prevalence of various types of citations in the methods sections of articles in highly cited journals, including shortcut ones, (b) examples of what happens when shortcut citations are followed and (c) a review of journal policies. This is followed by a reasonably extensive discussion focused on (d) guidelines on how to use shortcut citations.

I generally agree that this is an interesting structure, as it (a) documents the phenomenon, (b) evaluates to what degree it represents a problem, (c) inquires what is being made to address it and (d) suggests additional measures. The weakest link in the chain, however, seems to be point (b) (i.e. measuring the impact of the problem), as I am not sure the case studies provided are enough to quantify this. I will try to make this clear in my major points below.

Main concern:

- While the numbers of articles and citations in the first section of the study are probably sufficient to provide an overview of the use of citations, the 15 articles included as case studies in the second section are not. The authors seem to acknowledge this limitation, as they refrain from making a quantitative synthesis of these articles. That said, this leads this section of the manuscript to fall short in accurately presenting the importance of the problem. 

Although I found the visualization for each case study provided in Fig. S2 interesting, I would doubt that most readers will really make the effort to go through each one of them, much less be able to synthesize the data in their own heads to reach meaningful conclusions. Thus, I would strongly recommend that the authors provide some kind of quantitative synthesis of the problem in this section (i.e. What percentage of shortcut citations can ultimately be traced to the original reference? What's the average number of steps? What percentage is behind a paywall? What percentage reaches a dead end or an insufficient description?).

I note that 15 articles are probably too few for this purpose, and that the sample of articles in which citations are followed would have to be expanded. Thus, I would recommend that the authors perform a sample size calculation to reach the number of citations/articles that can provide reliable estimates within a given confidence interval. For this purpose, it's worth noting that it would be desirable to perform synthesis both at the level of citations (i.e. what percentage of citations in the sample can be traced?) and at the level of articles (i.e. what percentage of articles in the sample have at least one untraceable citation?), as citations within a single article should not be considered as fully independent units when it comes to representing the whole population of citations. Thus, using articles as units for the purpose of sample size calculation might be the better option.*

**Author reply:** We appreciate the reviewers request to expand the case series. When designing this project, the first proposal was to do a large version of the case series study. We quickly discarded this idea as it was not feasible. This study design would require a very different sampling frame and research team – to determine whether a method is described in enough detail to reproduce experiments, one must be intimately familiar with the details of the method described. One would need to conduct these assessments in a specific subfield or research topic and have a team that is knowledgeable of a broad spectrum of methods used in that field. Furthermore, differing experience levels introduce subjectivity – the level of detail needed by a researcher who is new to the field might be quite different from the level of detail needed by a researcher experienced in performing similar techniques. Additionally, researchers might reach different conclusions about whether the level of detail provided was adequate if they actually had to do the experiment, and had concrete information about whether it worked or not.

We believe that the information requested by the reviewer is best obtained through reproducibility projects, where research teams have had to go through papers and shortcut citations in detail to reproduce experiments. These teams will already have concrete information on what methodological information was missing. One could review these records to determine whether data were missing due to an inadequate description in the main paper, or in shortcut citations. One could also quantify the frequency of the types of problems that are introduced by the existing case series. We would be happy to collaborate with reproducibility projects on conducting these analyses in future studies, however this work goes beyond the scope of the current study.

We argue that the current study makes a significant contribution to the literature, for many reasons already recognized by the reviewer. To our knowledge, this is the first systematic assessment of shortcut citations. We provide a set of classifications that helps researchers to understand why authors use citations in the methods section, and offer initial data about the frequency with which shortcut citations are used across a spectrum of life sciences fields. We demonstrate some problems that can occur when using shortcut citations, while acknowledging that we cannot say how often these problems occur. We quantify the frequency of journal policies that may ameliorate or exacerbate problems caused by shortcut citations. Finally, we provide recommendations for responsible use of shortcut citations based on the results of our systematic assessment. No paper answers all important questions that could be asked about a particular research question. We believe that it is important to consider what the paper adds, relative to what is known from previous research.

***Reviewer #1 Comment:***
*- The categorization of scientific fields is somewhat strange: most people would probably consider neuroscience is a subfield of biology, so presenting both as separate categories may puzzle some readers. I understand that this is a consequence of the JCR categories used, but making this clearer from the start (e.g. "examine the use of shortcut citations in neuroscience, biology and psychiatry journals in the abstract) and perhaps referring to the biology journals as "general biology" would help to avoid confusion.

Still on this point, the selection of fields is narrow and ad hoc. I understand that this is a limitation posed by the authors' own expertise, but it is nevertheless one of the main weaknesses of the manuscript. Thus, the narrow range of scientific fields examined should probably be mentioned in the limitations section.*

**Author reply:** As one can see from the journals lists provided in the supplement (Tables S1-S3), the content covered by the neuroscience and biology fields is very different. As the field names come directly from the JCR categories, which was the sampling frame for the study, we do not want to change them. However, we have added a sentence highlighting the difference between the neuroscience and biology categories and referring readers to the journal lists for confirmation.

“The neuroscience journals examined generally focus on biomedical neuroscience, which may include basic science, translational and clinical research (Table S1). The biology journals examined publish a wide range of general biology research and are not exclusively biomedical (Table S2).”

We agree that all fields examined fall within the life sciences, however we would argue that our selections cover many different types of research in this domain. We have addressed this in the limitations:

“The three fields examined are within the life sciences and results may not be generalizable to other domains. The fields and journals selected do cover a range of research types, including non-biomedical (biology) and biomedical (neuroscience, psychiatry) research. While psychiatry research is predominantly clinical, neuroscience includes basic, translational and clinical studies.”

***Reviewer #1 Comment:***

*- Even within this relatively narrow sample of fields, the kinds of methods that deserve a protocol probably varies a lot: I'd guess that psychiatry journals include a lot of surveys and instruments, while biology and neuroscience might have predominantly wet lab protocols. It would be interesting if somewhere in the paper (possibly in the example cases provided) we could get a feeling of what kind of "protocols" we are talking about, even if only in a general sense. If quantifying/classifying them is not feasible, at least some illustrative examples could be provided. Are we talking about methods to quantify proteins? Scales to measure depression? Electrophysiology setups for rodents)? The citation culture probably depends a lot on the particular method, so the whole discussion sounds a bit disembodied without touching on this point somewhere.*

**Author reply:** Categorizing the types of methods and protocols used was beyond the scope of the current study. This was a journal-based sample; hence the types of methods used was extremely variable. We have briefly elaborated on this in the discussion.

“While it was not feasible to collect data on the types of methods used, the study team’s subjective impression is that the papers examined covered an extensive range of methods used in each field. The biology journals published both biomedical and non-biomedical research, including field studies, computational research and laboratory studies. Neuroscience and psychiatry journals had a strong biomedical focus. Psychiatry research is predominantly clinical, and resources cited as shortcuts often included diagnostic guidelines and surveys. Neuroscience included basic, translational and clinical studies.”

***Reviewer #1 Comment:***

*- Why are only minimum/maximum numbers of citations within shortcuts and the youngest/oldest citation coded? This looks like an approach to simplify data extraction, but it ends up providing very limited information (i.e. especially if there are many citations per paper, the oldest and youngest ones give very little information on the actual range).

Moreover, this ends up making data visualization in Fig. 3 much less intuitive than it could be (i.e. it would clearer and more informative to provide the full range of citation ages). If the authors could provide the full ranges (although I'm not sure that this is feasible), this would likely strengthen the paper. If not, I'd reconsider whether Fig. 3 should be included in the main results, as I don't think the results as displayed say much about the sample of citations as a whole.*

**Author reply:** We are a bit confused by the reviewer’s comment that the information about the minimum/maximum number of citations per shortcut or oldest/youngest does not accurately capture the range. This information exactly captures the range, which is defined as the difference between the minimum and maximum; we used this approach to bracket the extent of the problem.

We assume that the reviewer’s concern is that we have not fully captured the distribution. Notably, there are very few papers where the maximum number of cited resources in a shortcut is more than 2; therefore, obtaining more information about the data distribution for this variable will only provide information about papers in the tail of the distribution. We have added this information to the dataset and updated the visualization.

***Reviewer #1 Comment:***

*- Some points in the case series description and discussion mention that some references "provided a description that was no longer state-of-the-art" and that this may be a problem. I don't really get the idea here: methods citation are supposed to provide an accurate description of what was done in a study, not of what's the current state of the art of the method. In this sense, descriptions shouldn't age badly or become "not-state-of-the art".

I understand the concern that a very old shortcut citation raises suspicions that it might not really describe what was done in the paper (as it may be likely that no one uses certain methods in exactly the same way after 50 years). But if this is what the authors meant, this should be stated more clearly, as it is not really the impression that comes out of reading these passages.*

**Author reply:** The reviewer is correct that the concern with older shortcut citations is that they are less likely to describe methods that are currently used. We have updated the phrasing to reflect this. We thank the reviewer for helping us to clarify the manuscript.

***Reviewer #1 Comment:*** *In the same vein, mentioning in the discussion that "supplemental methods cannot be updated" is technically correct, but is not a limitation in terms of making methods sections reproducible (which seems to be the point of the paper). For the purpose of methods description, whatever was used in a paper should remain static, even though the method may evolve in subsequent study.*

**Author reply:** The paragraph highlights a variety of problems with supplemental methods, which introduce barriers to reproducibility and reuse. We updated the text regarding the final point, on inability to update supplemental methods, as follows:

“Fourth, supplemental methods cannot be updated after publication; hence, this format is not useful for sharing updated versions and tracking the evolution of protocols within and across research groups. This is not a problem when investigators’ goal is describe their methods for a completed study. Methods, however, are constantly evolving and are one of the most useful outputs that researchers create. Given this, it is more efficient for research teams to use platforms that allow versioning and forking when sharing research procedures, and cite the version of the method that was used for a particular research study.”

***Reviewer #1 Comment:*** *- In terms of data sharing, one thing I could not find in the manuscript or in the OSF was the DOI and title of the articles used as case studies in Fig. S2. I may have missed it, but as there was no folder for the case series section I didn't know where to look for it. As this seems important for reproducing the findings, this list should be provided somewhere (possibly as a document within the OSF) and cited within the text and legend to figure S2.*

**Author reply:** While we routinely share data from our meta-research studies, we historically do not publicly share data from studies that could be seen as highlighting specific authors, institutions, etc. as examples of bad practice. This is especially important when we are proposing new practices which are not yet widely accepted and will not have been known to the authors at the time their paper was written, as is the case in this paper. The results for the case series studies fall into this category, as they show citation chain maps for a small number of individual papers. We can share the DOIs for those papers privately with those who request them. We used a similar approach in our previous paper examining statistics education requirements for PhD training programs (<https://journals.plos.org/plosbiology/article?id=10.1371/journal.pbio.1002430>).

In contrast, data sharing is not a concern for the other two sub-studies, as the paper presents aggregate data showing practices across many papers and journals in three fields. Readers have no reason to focus on any specific paper in the dataset. Individuals who do chose to focus on specific papers in the dataset can interpret the data in light of the aggregate findings.

***Reviewer #1 Comment:***

*Minor concerns:

Introduction:
- The correct name of the project mentioned in the first paragraph is Reproducibility Project: Cancer Biology (not "for Cancer Biology").

- "This risk of bias for randomization sequence generation and allocation concealment was unclear…" - this sentence seems odd (in particular the "This" at the start), please revise the wording.*

*Figure 1: -The second "readers" can be omitted from the third sentence of the figure legend.*

**Author reply:** We have corrected these points – we thank the reviewer for helping us to improve the readability of the manuscript.

***Reviewer #1 Comment:***

*Figure 1:
- Isn't the methods section a viable alternative for sharing details needed to reproduce experiments as well? While I agree that in many cases a separate protocol may be a better option, that depends on the length of detail that is needed, which will vary greatly depending on the method. Therefore, I would argue that the methods section should be included as an option in the figure - saying that the information "should" be shared in a separate document sounds overprescriptive.*

**Author reply:** We have updated the figure to reflect that very simple methods that are easy to explain and reproduce may be described in the methods section, whereas protocol repositories and methods or protocol journals may be better for other methods. Note that this unfortunately introduces a very subjective element to the figure; what one considers to be a very simple and easily explained method is highly dependent on level of experience and other factors.

***Reviewer #1 Comment:***

*Methods:
- Instead of citing the full OSF page for "protocols, data and code for the prevalence study and journal policy studies" using a single link, wouldn't it make sense to cite a specific DOI for each of these resources? The same thing hold for points in the text in which specific resources are cited (e.g. "The full search strategy is available on the OSF repository" could point to a direct link to the search strategy rather to the full OSF page).
I think this is optional, as the Readme files in the OSF are clear. But providing specific links to each resource would be more consistent with the authors' recommendation of providing pages for book citations, for example (in the sense of sparing the reader the trouble to search for a resource within a larger space).*

**Author reply:** We thank the review for this suggestion, however we are concerned about the longevity of links. If OSF were to change anything about their website naming structure, links to individual items could all result in 404 errors. This would leave readers with the impression that materials are no longer available. The DOI and main repository link are more robust and should be preserved long term. We have therefore used the file naming convention and folder structure to help readers to efficiently locate information within the repository.

***Reviewer #1 Comment:***
*- What is meant by "top journals" exactly? Are those the ones with the highest impact factor in the JCR in their specific fields? Although this would be my guess, it is not clear from the description.*

**Author reply:** The reviewers’ interpretation is correct.

***Reviewer #1 Comment:***
*- The data on whether papers were related to SARS-Cov2 sounded rather gratuitous, as Covid-19 was not mentioned anywhere in the introduction. If this data is to be kept in the paper (I personally don't think it adds much), the rationale for extracting this should be mentioned somewhere.*

**Author reply:** Early in the pandemic there was a large wave of COVID-19 papers. At the time, the scientific was very concerned that the quality of COVID-19 papers might be lower than for other types of research due to the speed at which COVID-19 studies were being conducted and published. We included this variable so that we could address this potential concern. We have noted this when describing these methods.

Our dataset included vary few COVID-19 papers; therefore, we did not feel the need to address this question.

***Reviewer #1 Comment:***

*- Though this eventually became clear, I initially had a hard time to understand what was meant by "number of citations per shortcut". This could be made clearer when this variable is first introduced.*

**Author reply:** We have adjusted the language to “number of resources cited in a shortcut citation”.

***Reviewer #1 Comment:***
*- The description of a probable shortcut states that "additional details are not provided in the following sentences or elsewhere in the methods sections". But what happens if the method is fully explained outside of the methods section (i.e. in the supplementary material or in a repository)? I was unsure how these cases were classified, so it's probably worth commenting explicitly on it.*

**Author reply:** References to supplemental material were not evaluated in this study, as supplemental files are not citations and do not appear in the reference list (see definition of a citation in the protocol). We have added this information to the methods section.

If the authors cite a protocol deposited in a repository to explain their method, this is a shortcut citation. It does not matter whether the cited resource is a paper, a manual or guideline, something deposited on a repository, or something else. If the authors cite an external resource which appears in the reference list, instead of fully describing the method, it is a shortcut citation.

***Reviewer #1 Comment:***
*- Electronic searches were performed using the terms "[journal name]", "journal citation reports ranking", "author guidelines", "journal policy", and "impact factor". I don't quite get what this search means to achieve. Why would one need to search for "impact factor" to look for policies?*

**Author reply:** Abstractors used the term impact factor as a tool to verify that they were searching the website for the correct journal.

***Reviewer #1 Comment:***
*Figure 2:
- The different areas have different mean numbers of methods citations per paper (being somewhat higher in Biology). Thus, showing the results for different categories in percentages as in Fig. 2A may cause misleading impressions - although there are still less "How" citations in Biology than in Neuroscience or Psychiatry when measuring absolute numbers, the actual difference is smaller (while that in "Who or what" citations is even larger). Having the bars represent absolute numbers (possibly still displaying the percentage within the bars) - with overall longer bars for Biology - would likely provide a more accurate impression of what's going on.
- It took me a while to understand the right panel in Fig. 2B. While the fact the two sides of the violin plots represent different data eventually becomes clear, wouldn't it make it easier on the reader to break the information for probable and possible citation into separate plots (especially as the left panel uses symmetric violin plots)?*

**Author reply:** Presenting absolute numbers instead of percentages in Figure 2A would be misleading, as the number of papers assessed differs substantially across fields. We chose to use percentages to express citation behaviors for each field as a whole. The distributions for the number of citations in the methods section per paper are skewed; median values show that biology papers, as a whole, cite 2 more papers in the methods than neuroscience and psychiatry papers. The difference in biology is partly driven by STAR methods tables, which some journals use to show what materials were used in each paper. These tables sometimes contain citations, which are always classified as who or what citations to give credit or specify what was used. We added a supplemental figure (Figure S2), which visualizes the data for each category on a per paper level, using absolute numbers rather than percentages.

We updated the legend to clarify that data for possible vs. probable are presented on either side of the violin plot. We prefer this to separate plots, as it reminds readers that papers contain both possible and probable shortcut citations.

***Reviewer #1 Comment:***

*Tables S5 and S6:
 - Can't the information in these tables be included in the legend for Fig.2 and Fig.3 (as it is relatively short and essentially synthesizes the data in the figures)? This is optional, but would leave the information in one place instead of creating a lot of supplements.*

**Author reply:** The data from Table S5 has been added to the Figure 2 legend. Table S6 is quite complex and not easily incorporated into a figure legend.

***Reviewer #1 Comment:***
*Figure 5:
- Are the categories in Fig.5A and 5D mutually exclusive? It would seem to me that a journal could encouragd providing sufficient methodological details both in the author guidelines and as policy, and that they may encourage sharing methods in more than one place (i.e. repository or supplemental files). This is likely worth commenting on in the legend.*

**Author reply:** The categories in 5A are mutually exclusive. Journals may recommend sharing in detailed methods or protocols in more than one place in 5D; we have clarified this in the legend.

***Reviewer #1 Comment:***
*Discussion:
- I don't think the Germany and California examples mentioned in Box 1 are needed: there are plenty of places of the world with much worse access, and these particular examples are not particularly representative of difficulties faced by the world at large.*

**Author reply:** We removed these examples.

***Reviewer #1 Comment:***
*- While I agree with the recommendation to "make all methods publications open access", I don't think that there's any particular reason why methods papers are different from the rest of science (in the sense that they should be open access), so I'm not sure the recommendation really belongs here.*

**Author reply:** We agree that there are substantial benefits to making all papers open access and that doing so would eliminate the problem. While there is some progress, the problem will not be solved any time soon.

We do think that there is a special case for making methods and protocol papers open access and moving methods sections in from of the paywall. Shortcut citations are unique in that they replace a part of the methods section of the paper; readers will need to access these resources to obtain details of the study methods. This is not necessarily true for other types of citations. As we outline in the paper:

“*Methodological shortcut citations replace a section of the methods. Detailed methods are essential for those seeking to implement the method. We therefore propose that resources cited as methodological shortcuts should meet higher standards than materials cited for other purposes.*”

We have adapted this recommendation to also include moving methods sections of closed access papers in front of the paywall, in line with the PRO-MaP (Promoting Reusable and Open Methods And Protocols) recommendations.

***Reviewer #1 Comment:***
*- The discussion about copyright issues described in the list of recommendations is long for an item in a list. Thus, it probably would fit better in the main text or in a box.*

**Author reply:** While it is slightly longer than other points, we would prefer to keep the copyright content where it is as it provides context that is directly relevant to the recommendation. This is a common objection that editors and journal staff raise when justifying these policies. Many authors tell us that they’ve had to make methods section less detailed or cite papers due to threats of action over copyright violations. Putting this information in a different place increases the likelihood that this point would be missed. We would welcome the editor’s input.

***Reviewer #1 Comment:***
*Table S7:
- I get the feeling that Table S7 would read better if lines and columns were reversed (i.e. methods as lines, features as columns), but it may be a matter of taste.
- Why are supplemental files and protocol journals deemed static while shortcut citations are not? This does not make much sense to me.*

**Author reply:** Shortcut citations are not denoted as static or dynamic in the table; there is simply a notation that authors must describe modifications if the methods described in the shortcut citation differ from those used in their own research.

*- I'd say supplemental files would generally be expected to have been peer reviewed. I agree that this is likely not always the case, but that probably depends more on the reviewer than on the journal (e.g. I don't know of journals that explicitly exempts supplementary material from the peer review process), so I'd remove "depending on the journal".*

**Author reply:** We changed this to “The quality of peer-review for supplements may be variable”

*- The comment "protocols remain available over time" made for repositories stands for all categories - it makes sense when comparing a protocol repository to a lab notebook, not to the other forms of describing protocols. Thus, I'd probably not include it as an advantage here.*

**Author reply:** We removed this.

*- I'd argue that both shortcut citations and supplemental files are "findable" for whoever's reading the paper (which is likely what matters here), so I'd be inclined to remove this category.* 

**Author reply:** Many methods are of interest to people who would not otherwise read the paper; if we want to take advantage of the potential benefits of open research, we need to modularize research outputs and create search engines that easily allow researchers to find methods, data and code that are relevant to their own work. There are many cases where researchers are unaware of established methods that might be useful for their own research because those methods are published in papers from fields or subfields that they do not normally read. In contrast, searching for keywords on a protocol repository or using a protocol search engine would help readers to find this information and facilitate transfer of methods across fields. Supplements are not indexed and they are not findable; relying on them to share methods is extremely inefficient.

*- Clinical journals are not the only one to publish protocols as articles (the systematic review community has a tradition of publishing protocols, for example).*

**Author reply:** We have amended this as follows: “Some journals publish study protocols for clinical studies, systematic reviews, etc.”

***Reviewer #1 Comment:***

Figure 6:
- In the last no/no option, describing the method in the main text (if it is simple enough to fit) should also be included as an alternative. 

**Author reply:** We have adjusted the figure as suggested.

***Reviewer #2 Comment:***
*------------------
Disclaimer/COI: 
------------------
As disclosed to the editors, to be fully transparent I'll include this information here: I currently work at* [*https://protocols.io*](https://protocols.io) *and therefore have a very obvious positive bias about the value and importance of protocols and methods sharing as a part of the scientific research process. I have also participated in some working groups with Tracey Weissgerber. And less relevant here but in the interests of full disclosure I was previously employed in an editorial capacity at PLOS Biology.

------------------
Overall View and Recommendation: 
------------------
This is an important meta-research study into the status of quo of how methods and protocols are cited in the scientific literature. The authors perform a detailed and thorough investigation of how researchers cite methods, the issues encountered by a reader who consults citations to understand methods that were used, and protocols followed, and how journal policies differ with respect to requirements or encouraged behaviour relating to methodological reporting.

This is important because methods sharing has lagged behind expectations around the sharing and availability of other research outputs like data, code, articles themselves etc. And yet insight into methods is critical for interpretation and reuse of data.

With Minor Revisions I would recommend this for publication in PLOS Biology. I don't have many comments and the first one is my subjective take on things, so will leave to editors/authors to decide whether to make changes or not in response.

------------------
Comments:
------------------

1.      My one main comment is possibly a bit subjective, and the authors may have considered this already, but for the categories shown in Table 1 (for the reasons for citations in the methods sections), the difference between 'Who or What' and 'From where' isn't very clear. Both really seem to me to be ways to give credit: 

- for the 'Who or what' for the wording used is: "give credit to the group that created the method, tool, resource or substance, …" 

- and for the "From where" category the wording is that "The citation is used to show where a substance or organism was obtained from", and the example given is a lab as source of a mouse - this seems to be as much of a "Who" as a "From where".

Both categories include "substance" and seem to encompass resources/tools/materials - a mouse model could as easily fall into the Who or What as a Where from, I think these could easily be the same category. 

In the results the numbers for the "From where" are quite small, I wonder if these categories might be better off all rolled into one? I appreciate that this would involve a rework of how results are reported and some of the assessment, but worth thinking about whether these are distinct categories (you could still show sub-types within as you already do for the Who or what section in Fig 2).*

**Author reply:** We understand that other teams using this protocol in the future may choose to combine these categories. As they were not combined in our original protocol, we have chosen to present them separately in accordance with the protocol.

***Reviewer #2 Comment:***
*2.      Very minor: Under the description for Case Series on P9, it's noted that an assessment was made to determine if the methodological description was "adequate" - I can infer what is meant by adequate, but it might be helpful to add a definition of what would qualify as adequate.*

**Author reply:** We have added the following sentence: “Descriptions might be judged as inadequate if they were clearly missing essential details that would be needed to implement the methods.”

***Reviewer #2 Comment:*** *3.      Very minor pedantic comment: The order of Neuroscience / Psychiatry / Biology in the Key for Figure 2 is different from the order that the categories are shown in for each of the figure parts where they're always shown in the order Neuro / Bio / Psych. Suggest authors switch the key order to be the same as the figure panels.*

**Author reply:** Thank you – we have fixed this error.

***Reviewer #2 Comment:*** *4.      Journal policy assessment is interesting to read through, but I wonder if the authors might care to suggest an ideal wording for a journal policy that could then be adopted by journals who want to do better. Even if worded as encouragement rather than mandate this might very valuable and appreciated (of course as with all these things it may also be contentious, so for authors to decide if they want to go there or not…). Another option could have been to identify if there were any exemplary journals out there already, with or without the wording that they use?*

**Author reply:** We were very interested in this suggestion and debated this extensively, however ultimately decided not to suggest specific language for policies. Often policies are set by the publisher; hence, wording is very similar for all journals published by the same publisher (e.g., Elsevier, Taylor and Francis). Hence, we have fewer policies than one might expect. The PRO-MaP project also recommends policy changes, and we’ve found in consultation sessions that editors prefer a less prescriptive approach. Many find clearly articulated principles most valuable. As they already have policies related to some of these things, they are likely to adapt their existing policies instead of introducing something new. Finally, our recommendations would be based on theory rather than evidence - we don’t know what works. Existing evidence suggests that without enforcement, changes in policies have little effect on author behavior. Publishers rarely choose to invest resources in enforcement. Meta-research studies clarifying whether policies and enforcement mechanisms are effective will be needed.

***Reviewer #2 Comment:***
*5.      While I'm well versed in what forking means for a methods-sharing platform like protocols.io, personal experience has made it clear that to a lot of researchers the terminology of forking it not as clear as it could be. The authors should consider including a short definition/description of forking so that there is an easier understanding of the value of being able to easily fork a protocol (maybe using an example e.g. easy creation of a similar protocol where tweaks are needed in order to apply it a different organ or cell type, or to use a new piece of equipment, and how this is facilitated if you can start with a forked copy of an existing protocol so that protocol creation from scratch is not needed).*

**Author reply:** We have defined the terms versioning and forking in the text box where they are first mentioned.

***Reviewer #2 Comment:***
6.      I really like the citation pathway representations in Fig S2 - is it worth putting one example into the main text for a reader to see one without having to go to the Sup file. I think it's very illustrative of the entire article.

**Author reply:** Thank you – we have moved two examples into the main text (Figures 4, 5). All figures for the case series remain in Figure S2.

***Reviewer #3 Comment:***
 *Dear authors, 

I congratulate you all for this very important piece of research I had the privilege to review. Please enface my comments as a way to improve your manuscript and not as a criticism. Also, I am going with some doubts throughout the text I hope you may have the opportunity to explain that in details. 

\### Comments ###

1. Title: for my sense, the title should be more technically informative, specially in terms of design. Please use the Murad et al (2017) reporting guideline for meta-research studies to guide your writing.*

**Author comment:** Thank you for this suggestion. Including study design information in the title is common for certain study types (e.g. systematic reviews) and in particular fields (e.g. epidemiology and clinical medicine). This practice can be very useful in fields where researchers are knowledgeable about study designs and there is clear consensus about the names for the types of designs that are used. We worry that it may have unintended consequences in fields where readers are less knowledgeable (e.g., widespread misuse of “case-control study” to refer to cross-sectional studies that include a group of patients who have a disease or condition of interest, and a group of controls who do not have the disease or condition of interest).

In this particular case, specifying the design in the title is complicated. There are three different sub-studies and, as pointed out by the reviewer in subsequent comments, some of them do not clearly fall into established study design categories.

We have changed the title, albeit for different reasons. In conversations with researchers who read the preprint, we learned that some readers concluded that shortcut citations should never be used. This is not our goal; citing a protocol on an open repository is an excellent use of shortcut citations and should be encouraged. The implication of this study is that we need to encourage responsible use of shortcut citations. We believe that the old title was contributing to this misconception. The new title is more focused on responsible use of shortcut citations.

Revised title: Taking shortcuts: Identifying strategies for using methodological shortcut citations responsibly

The guideline referenced focuses on meta-epidemiologic studies – according to the definition provided by the authors, these are systematic reviews or meta-analyses that use data from clinical studies to address questions related to the methodology of those studies. As such, these guidelines still contain some items that are not relevant to the current study, which we have not addressed. As noted in the manuscript, some items (e.g. search strategy, abstraction protocol) are provided on the repository, rather than in the paper itself. Our study was exploratory, as we are not aware of prior research on this topic; therefore, we present goals, but not hypotheses. As this guideline has substantial overlap with PRISMA, we feel that we have addressed relevant items from the guideline either in the paper or on the repository, often at length.

***Reviewer #3 Comment:***

*2. Abstract: it is a matter of style, but for the vast majority of the times, a structured abstract works better for readers (and referees) than a non-structured one. I strongly recommend a structured abstract for your manuscript, although not being an exigence of PLOS Biology journal.*

**Author comment:** We agree that the structured abstract has many benefits. Unfortunately, the editor has noted that it is not used by PLOS Biology.

***Reviewer #3 Comment:***

*3. Main text: 
3.1. Structure of sub-studies: 

- I'm up to believe your first study does not fit into a classification of a prevalence study (indeed, there is no prevalence study among study designs, but cross-sectional studies). To gather a true prevalence, your denominator should be all manuscripts of the studied population, which is not true. What you have in hands are relative frequencies. Therefore, for my sense, to claim fhe first study as a prevalence study is not correct at all. I would recommend "bibliometric analysis" OR "cross-sectional analysis". So, it serves also when presenting results - you cannot sustain prevalences in your report, rather than frequencies. It should be switched.

- As for your second study, I won't classify as a case series study as well. This is a clinical classification, although I understood the analogy. A term that is well accepted in the meta-research literature is a "cohort of studies", which I recommend for. 

- Finally, your third study also does not fit into your classification for my understanding. What is a "journal policy study"? When you are cross-sectionally analyzing journal policies (which you did, not longitudinally), it is a cross-sectional study, but your unit of analysis are not patients - rather, studies. Then, I strongly suggest to revisit it.*

**Author reply:** We have adjusted the names of the studies to reflect what is being examined; then noted the design within the methods description of each study. This will make it easier for readers to differentiate between the three sub-studies.

Prevalence study was changed to “Methodological citations study”

Case series was changed to “Shortcut citation chains study”

Journal policy study was unchanged

***Reviewer #3 Comment:***

*3.2 Stratification of studies

- It wasn't clear for me. Did you take all of your studies, split them into quintiles of their JCR, and randomly selected a fraction of studies of each quintile? If yes, it does make sense for me, but I would love to hear from you if you think that JCR may be a proxy of editorial policies (word limit) and quality of reporting; and, how the potential role of the study design and the nature of intervention were accounted for? I explain: RCTs and pre-clinical studies usually requires more detailed methods explanation than other designs; or, for a given discipline (Psychiatry, for example), a trial published to pursue an FDA/EMA market approval is completely different of a trial in which the intervention is physical activity, for example (potentially). This should be highly acknowledged - if not possible to control, acknowledge as a limitation in your discussion.*

**Author reply:** We are not sure which study the reviewer is referring to, which makes it difficult to answer this question. In the methodological citations study (study 1), we selected the 15-20 journals with the highest impact factor for each field, as described. Journals that did not publish original research were excluded, and replaced with the next journal on the list until we reached the desired number of journals (15 for biology, 20 for neuroscience and psychiatry). We then assessed all original research papers published in those journals during March of 2020. We do not like impact factors – the problems with this metric are widely known and extensive. However, choosing journals at the higher end of the impact factor spectrum addresses concerns from some reviewers that any poor practices observed may be due to the inclusion of “low quality” or predatory journals. Using a larger number of journals reduces the likelihood that findings may be unduly influenced by unique features or policies of a specific journal or imprint.

For the journal policy study, we assessed all journals, regardless of impact factor.

We agree with the reviewer that some study types may have traditions of better methods reporting, or more urgently recognize the need for strong methods reporting, compared to others. However, the fact that a field has less regulation around methods reporting doesn’t mean that reporting of high-quality methods is not important. The sample includes many different types of studies and abstract data on study type was not feasible. Our subjective impression is that the proportion of clinical trials was small, and the proportion of clinical trials that would lead to a FDA/EMA market approval was even smaller. Furthermore, one might expect that the authors would use a shortcut citation to refer readers to the study protocol rather than attempting to describe all methods in the paper. Future studies may investigate whether study type influences shortcut citation use or other aspects of methodological reporting quality, however this was beyond the scope of the current investigation.

***Reviewer #3 Comment:***
*3.3 Results

- Personally, I think that is clearer for the readers to be redundant in the main body and figures/tables when talking about results. I felt lack of clarity in results, specially in the explanation of the violin plots. Is that possible for you to use redundancy and write the results presented in figures/tables as whole text as well? Quite an analogous of the methods shortcut. We can't presume that all readers do know to interpret three-factors (or more) plots easily and nowadays to make science communicable to peers is more imperative than ever.

- I liked the way you structured the logical of presenting results. They are suitable in a timeline manner and make sense intrinsically.*

**Author reply:** We thank the reviewer for helping us to clarify the manuscript. We have provided some additional explanation for how to interpret the violin plots in the figure legend, as requested by reviewer 1.

We are not sure exactly what information the reviewer is looking for when requesting more redundancy between the figures/tables and text of the results. Notably, we have not re-stated the specific numbers shown in individual figures. We have instead commented on general patterns that are visible in the figures and tables. In initial submissions for previous papers, we had repeated numbers from the figures or tables in the text. Reviewers commented that this made the results section extremely long and tedious, and they lost track of the main findings amid the detailed reporting of exact values. Past reviewers strongly preferred the strategy that we have used in the current paper.

***Reviewer #3 Comment:***
*3.4 Discussion

- I liked as well how you dedicated plenty of space for the future recommendations. We definitely should bring solutions rather than accuse the problems. This is easy. I congratulate you as a whole about this. My only minor point is that I felt some explanation how you determine "why" shortcut citations were used if a mixed-methods study was not conducted. Is it an inference (not statistical)? Did you gather any proxy? For my sense, this is a very difficult question to answer based only in a manuscript screening.* 

**Author reply:** This study did not include mixed methods; types of citations were categorized based on review of each paper using the general descriptors for each category of citation type, as outlined in the protocol (e.g. explain a method, provide context/information or refer to previous result). The question words were added during the writing process, as we have found that they make it much easier for readers to remember the categories and quickly understand what they represent (e.g. citations in the “Explain a method” category answer the question “How was this method performed?”). In the limitations section, we comment the challenges of categorizing citations and inferring authors’ intent based on the text provided.

“Data examining the reasons for citing a paper in the methods (Figure 2a) should be viewed as a rough approximation. Abstractors were making distinctions about the reasons for a citation that the authors themselves likely did not make when inserting citations into their methods sections. Small changes in the wording or position of the reference could alter the categorization, as could variations in reader expertise.”

***Reviewer #3 Comment:***

*4. Final Considerations

I appreciated this piece of research. Personally, in times in which the discussion of journal standards for submission and etc. is very pertinent. I advocate for formats like this, although I disclose I don't recognize it as a potential conflict of interest to review this manuscript. My central final question for you is: among your all recommendations, how to deal *pragmatically* with the problem of shortcut methods citations while culture changing doesn't finish your cycle (which takes a lot)? We do know that hybrid journals will be still reluctant to expand room for words; we do know that, still nowadays, researchers are afraid to deposit supplemental material and/or data (by the way, congratulations for the data-sharing practice) or even don't know how to do it if they want either - and the list goes on. Theoretically all recommendations make a lot of sense for me, but the problem seems to be urgent and a pragmatic solution needed. Do you have something in mind?*

**Author reply:** This is a complex issue and there are no simple solutions. The PRO-MaP (Promoting Reusable and Open Methods And Protocols, <https://osf.io/x85gh/>) recommendations offer solutions for improving methods reporting in general, by highlighting actions that researchers, researcher institutions and departments, funders and publishers and editors can take to improve methods reporting in life sciences publications. This includes specific recommendations related to the use of shortcut citations.

Pragmatically speaking, systemic solutions are needed and they likely need to be implemented at the journal level. Unfortunately, journal policies often aren’t followed. Enforcing policies requires resources and few publishers will provide these. Automated tools can be helpful to detect common problems, however integrating them into legacy publishing systems is very costly and time consuming. Furthermore, detecting shortcut citations is a complex task and it would be difficult to train an algorithm for this. One thing that we likely can do is to train a tool to detect common phrases in the methods section that are regularly used in shortcut citations (e.g. briefly, as previously described). If we have a tool to detect such phrases, we could give authors brief reports noting that it’s likely that they have used one or more shortcut citations and outlining suggestions for using such citations responsibly. However, as >90% of papers used shortcut citations in all fields examined, the value added by the tool may be limited.

***Reviewer #4 Comment:***
*This paper reports an exploratory study looking at shortcut citations, an approach used to cite a previous paper instead of describing the methods in detail. The authors found that citation used in methods sections were common with methodological shortcuts common among the 3 fields investigated. Case studies were also conducted to examine how methodological information was able to be located (or not) for several papers. Finally, the paper examined the journal polices around making methods sections complete and reproducible. Overall, this paper highlights a major challenge in how scientific information is communicated, specifically around the methods used in published research findings. The paper is very detailed and clearly communicated. My suggestions below are recommendations to the authors to consider when revising their paper.

(1)     While this paper focuses on the way authors use citations in methods for papers published during March 2020, do the authors have any way to help readers understand how this relates to how methods were reported in the past? That is, is this a new phenomenon or something that's been entrenched in scientific methods sections over the years? This is beyond the scope of the study, so my question is only if there are other papers on this topic that might be considered in the discussion. If not, it might be relevant to also discuss that it is unknown to the authors how these findings compare over time because of a lack of identified literature on the topic.*

**Author reply:** We are not aware of any prior research systematically examining the issue of shortcut citations; as such, we cannot comment on whether use is increasing over time.

***Reviewer #4 Comment:*** *(2)     Did the authors look at whether the short-cut citations were self-citing? That is, how common are short-cut citations to one's prior work?*

**Author reply:** We agree that this is an interesting question, however it was not something that we examined in this paper. We have heard from researchers since posting the preprint that some researchers cite their own citations as shortcuts to increase their own citation counts, so this would be an interesting question for future study.

***Reviewer #4 Comment:*** *(3)     I believe the reason for why citations in supplemental method were not examined was because of a logistical constraint. The authors discussed their subjective impression that many supplemental methods lacked details or described specific details (e.g., list of primers). However, do the authors believe that shortcut citations also exist in supplemental methods and/or repositories? And if so, what potential impact would this have on how readers should interpret the findings reported in this paper? Is it a 'best case' scenario and that the number and incidence of shortcut citations is likely to be higher than what's reported here?*

**Author reply:** Some papers may also have used shortcut citations in supplemental files, however we were not able to quantify this as we lacked the resources to assess supplemental files. We have noted this in the limitations section.

***Reviewer #4 Comment:*** *(4)     While the authors did not look at completeness of reporting or other aspects of methods reporting, something that shortcut citations reminds me of is another practice that occurs in methods sections where information is not fully detailed, specifically using the phrase 'following standard protocol' instead of writing out or properly linking to the methods used, especially since what is 'standard' varies by lab. While this might be beyond the scope of this paper, the authors might consider how this practice is similar or not to shortcut citations and how this might impact the recommendations proposed.*

**Author reply:** Inadequately detailed methods can either be due to a lack of detail in the methods of the paper, or to a lack of detail in shortcut citations. We examined only the second problem, although the first is also a major issue. This was acknowledged in the limitations.

“This study focused on shortcut citations. We did not examine the completeness of methodological reporting when authors described a method without using a shortcut citation.”

This is another reason why data from reproducibility projects would be valuable – this would help us to determine how often details are missing from the methods section, vs. shortcut citations.

***Reviewer #4 Comment:*** *(5)     The authors might also consider this initiative (*[*https://elifesciences.org/inside-elife/c40ae742/elife-latest-request-detailed-protocols-easily-via-bio-protocol-integration*](https://elifesciences.org/inside-elife/c40ae742/elife-latest-request-detailed-protocols-easily-via-bio-protocol-integration)*) in addition to the one from PLOS One highlighted in the discussion.*

**Author reply:** Thank you for this suggestion. There are various publishers that have relationships with protocol journals (e.g. Cell Press and STAR Protocols). The reason why we highlighted the PLOS One Lab Protocols article format is that the protocol itself is a link to protocols.io, which offers versioning and forking. Whereas protocols published in journals can quickly become outdated, publications linked to repositories that offer versioning and forking allow readers to quickly see and access an updated version of the protocol. Bio-protocol does not offer these capabilities in their journal, although their preprint server has integrated versioning and forking.

***Reviewer #4 Comment:*** *(6)     Figure 6 has a typo in the bottom left circle - it should be 'that does not meet' instead of 'the does not meet'*

**Author reply:** Thank you! We corrected this.
