# Supplementary material for: Shortcut citations in the methods section: Frequency, problems, and strategies for responsible reuse
Source: PLoS Biol. 2024 Apr 2;22(4):e3002562. doi: 10.1371/journal.pbio.3002562 (PMC10986953; doi:10.1371/journal.pbio.3002562)
Supplement: Attachment — Submitted filename: Shortcut_citations_R2_ResponseToEditor_20240222.docx [file pbio.3002562.s012.docx]

**Response to editor**

We completed the following adjustments in accordance with the decision letter:

1. Updated the title
2. Adjusted the abstract to incorporate key findings that were not included previously
3. Incorporated links to the data repository in figure and table legends
4. Checked the references. One reference was removed as the link was no longer active and we could not find the cited materials.
